# *Pseudomonas aeruginosa* LasI/RhlI quorum sensing system controls protease-mediated autoaggregation behavior, cell envelope characteristics and extracellular proteome responses

**DOI:** 10.3389/fmicb.2025.1693814

**Published:** 2026-01-05

**Authors:** Albin Eriksson, Maria V. Turkina, Maria Ntzouni, Karl-Eric Magnusson, Elena Vikström

**Affiliations:** 1 Department of Biomedical and Clinical Sciences, Faculty of Medicine and Health Sciences, Linköping University, Linköping, Sweden; 2 Core Facility, Faculty of Medicine and Health Sciences, Linköping University, Linköping, Sweden

**Keywords:** *Pseudomonas aeruginosa*, quorum sensing, *N*-acyl-L-homoserine lactone, aggregation, cell envelope integrity, proteomics, virulence factors, homeostasis

## Abstract

Quorum sensing (QS) is an intercellular communication mechanism employed by the opportunistic bacteria *Pseudomonas aeruginosa* to regulate processes beneficial to the longevity of the community and related to its pathogenicity. The LasI/Rhl circuits of the quorum-sensing (QS) network operate through *N*-acyl-L-homoserine lactones (AHL) and appear at the top in the QS hierarchy. In natural habitats and host environments, bacteria exist and transit between different modes of lifestyle: planktonic single cells, suspended multicellular aggregates, and surface-attached biofilm communities. Using *P. aeruginosa* PA14 as a model, we determined the contribution of the master regulator LasI/RhlI QS system to multicellular community autoaggregation in liquid, ultrastructure and fitness characteristics of the cell envelope, and extracellular proteome responses, employing phenotypic assays, light imaging, high-resolution transmission electron microscopy and quantitative mass spectrometry-based proteomics and bioinformatics. Wild-type bacteria with a functional QS system were more effective in the protease-mediated autoaggregation than the *lasI-/rhlI-* mutant, lacking the production of AHL molecules and associated virulence traits. AHL-dependent communication impacted cell envelope characteristics, including ultrastuctural curvature and tolerance to membrane-damaging and antimicrobial agents. Moreover, the LasI/RhlI QS system perturbed the extracellular abundance of a total of 545 extracellular proteins during late exponential and early stationary growth phases. We allocated most of these differentially expressed proteins to the following large functional groups: metabolism; transcription and translation; transport and secretion systems; cell envelope integrity; redox processes; invasiveness and toxicity; and motility. Remarkably, approximately 95% of the extracellular proteome was upregulated in the *lasI–/rhlI–* mutant compared with the wild type, and these levels were restored to wild type-status when AHL was added. We observed a crucial contribution of the LasI/RhlI QS system to the protease-mediated community autoaggregation in *P. aeruginosa* PA14. Mechanistically, this was accompanied—through a sophisticated and multifactorial process—by differential expression of an array of components in the secreted proteome involved in both pathogenicity-specific and global readjustments in the homeostasis within the population. By fine tuning the LasI/RhlI system, *Pseudomonas* can regulate its pathogenic potential and long-term survival in different hosts and habitats.

## Introduction

The gram-negative bacterium *Pseudomonas aeruginosa* is omnipresent in natural habitats, particularly in environments influenced by human activity (Crone et al., 2020). As an opportunistic human pathogen, it ranks among the foremost agents of severe nosocomial infections across diverse tissues, with a pronounced incidence in immunocompromised individuals and those exhibiting impaired physical barriers. When acute infections are not fully eradicated, *P. aeruginosa* may transit to a chronic state that is substantially more aggressive, tolerant to drugs and antibiotics, and thereby demanding more advanced treatment (Tummler, 2022; Pont et al., 2022). The emergence of *P. aeruginosa* infections is associated with the definition of highly virulent, antimicrobial, and multidrug-resistant superbugs, being classified as the ESKAPE group of pathogens (De Oliveira et al., 2020) by WHO.

A principal feature of *P. aeruginosa* is its remarkable adaptability, including the ability to assemble into biofilms as surface-attached multicellular communities encased within a self-produced extracellular polymeric matrix. This milieu fosters coordinated virulence factor expression and confers enhanced survival within the host, escape from host immune defenses and antimicrobial agents (Breidenstein et al., 2011; Poole, 2011; Hoiby et al., 2010). This flexibility is also supported by intrinsic, acquired, and adaptive resistance mechanisms to multiple classes of antibiotics (Goodman and Lory, 2004; Breidenstein et al., 2011). Intrinsic resistance is mainly maintained by an altered permeability barrier formed by the cell envelope. There is a complex and synergistic interplay between its structural layers, lipopolysaccharide (LPS) patches and OprD family porins in the outer membrane (OM), and Mex active efflux pumps localized in the plasma membrane (PM), acting to exclude or facilitate the passage of drugs (Maher and Hassan, 2023; Moradali et al., 2017). Changes in bacterial envelope, e.g., via its composition and topography reflect fitness and adaptability not only of the envelope but of the whole bacterium (Lithgow et al., 2023). Furthermore, a lower level of antibiotic susceptibility is provided by the gene *ampC* encoding inducible *β*-lactamases (Maher and Hassan, 2023; Moradali et al., 2017). Additionally, *P. aeruginosa* is equipped with an extensive repertoire of destructive and toxic virulence determinants causing host cell death, modulating host–pathogen interactions and helping the bacteria evade immune clearance (Tummler, 2022; Pont et al., 2022).

The remarkable accommodation of *P. aeruginosa* is governed by the plasticity of its large and complex genome (5–7 Mbp) and supported by many regulatory genes and networks (Stover et al., 2000). Among them, quorum sensing (QS) plays a central role, enabling rapid and coordinated responses to environmental changes. It comprises intercellular communication driven by the synthesis and detection of diffusible signal molecules, enabling population density-dependent regulation of pathogenic traits (Mukherjee and Bassler, 2019). *P. aeruginosa* harbors at least three interlinked QS circuits—Las, Rhl, and Pqs—each exerting hierarchical and autoregulatory control over each other (Papenfort and Bassler, 2016). The Las and Rhl systems operate via *N*-acyl-L-homoserine lactones (AHL), synthesized by the LasI and RhlI enzymes as *N*-3-oxo-dodecanoyl-L-homoserine lactone (3O-C_12_-HSL), and *N*-butanoyl-L-homoserine lactone (C_4_-HSL), respectively. As the cell density increases, these AHL accumulate until they reach a threshold (“quorum”) that permits binding to their cognate cytoplasmic transcriptional regulators—LasR for 3O-C_12_-HSL and RhlR for C_4_-HSL—thereby activating the expression of hundreds of genes, out of which over 30% encodes virulence factors (Miranda et al., 2022; Schuster and Greenberg, 2006). Collectively, QS influences the expression of up to 10% of the *P. aeruginosa* genome and alters the abundance of more than 20% of proteins in major cellular compartments (Arevalo-Ferro et al., 2003; Nouwens et al., 2003; Hare et al., 2012). Specifically, QS controls the timing and production of extracellular compounds: destructive toxins, degradative enzymes, effectors that neutralize host defenses, biofilm matrix components, metabolic processes, iron-scavenging siderophores, and motility functions (Miranda et al., 2022). Moreover, there is accumulating evidence to indicate that QS cross-talk exerts modulatory effects on host cell functions through distinct signaling pathways (Turkina and Vikstrom, 2019).

*Pseudomonas aeruginosa* PAO1 and PA14 have been widely used to gain new knowledge on bacterial pathogenicity and the discovery of novel drug targets and treatments. While PAO1 and PA14 have both been successfully employed to model various QS aspects, some genetic and phenotypic similarities and discrepancies have been observed concerning secreted products, metabolism, chemotaxis, biofilm formation, and protective mechanisms. Thus, PAO1 and PA14 are modeling moderately and highly virulent characteristics, respectively (Grace et al., 2022). Based on observations on these typical strains, the Las and Rhl pathways, acting both hierarchically and cooperatively, are essential for the regulation of the Pqs circuit. The Las pathway is generally considered to be the top player in the QS hierarchy, since its activation is required for the regulation of the other QS circuits, the Rhl and Pqs. In addition, the Las and Rhl systems regulate each other. Thus, the Las circuit’s 3O-C_12_-HSL can induce increased expression of the *rhlI* gene, while the Rhl circuit’s C_4_-HSL increases the expression level of the *lasI* gene. Moreover, these two systems work cooperatively, where a combination of 3O-C_12_-HSL and C_4_-HSL is likely required for maximal effect (Deziel et al., 2004; Schuster et al., 2003; Miranda et al., 2022; Thomas et al., 2025). Along with the Rhl system, Las actively mediates QS signaling at the earlier stages of the exponential growth phase, while the PQS signaling pathway is becoming prominent later in the exponential phase and peaking in the late stationary phase (Choi et al., 2011). This timing and specificity ensure that different arrays of QS-controlled genes, transcriptome, virulence traits, and metabolic products are triggered and expressed to various degrees at appropriate stages of bacterial growth and during infections, which can tune bacterial fitness and adaptability in the specific social environment (Schuster et al., 2003; Nouwens et al., 2003; Dotsch et al., 2012; Davenport et al., 2015; Mellbye and Schuster, 2014). Moreover, strains with mutated *las* and *rhl* genes appear less virulent than wild-type bacteria in experimental models (Tang et al., 1996; Wu et al., 2001; Rumbaugh et al., 1999). The *rhlI* mutant infects animals as effectively as wild-type *P. aeruginosa*, while the *rhlR* mutant is attenuated (Mukherjee et al., 2017). Recent findings have highlighted a natural appearance of LasR-defective strains in both acute and chronic clinical settings (Deziel et al., 2004) and diverse environmental niches (Groleau et al., 2022). These strains tend to have compensatory mutations in other QS-related genes, such as *rhlI*, adjusting the levels of C_4_-HSL and restoring virulent phenotypes (Simanek et al., 2023). Further, community responses within the population and transcription regulators may modify the activation of *lasI,* and accordingly, 3-oxo-C_12_-HSL accumulation in LasR-defective bacteria (de Oliveira Pereira et al., 2023).

The lifestyle of *P. aeruginosa* involves transitions between natural habitats and human hosts. In either niche, dispersal planktonic single cells, suspended multicellular autoaggregates, and surface-attached biofilm multicellular communities appear. In clinical settings, like in cystic fibrosis lungs, aggregates, and biofilms are implicated in life-long lung infections that often lead to failure of the respiratory system (Bjarnsholt et al., 2013). In autoaggregation, bacteria of the same strain form multicellular clumps driven by interactions via self-produced autoagglutinins (Kragh et al., 2023). Generally, molecules of different classes can act as autoagglutinins: cell appendances (flagella and pili); other cell surface-associated structures (secretion system components, outer membrane proteins); and extracellular biopolymers (eDNA, polysaccharides and proteins). Moreover, changes in the cell envelope architecture and fitness, such as those triggered by environmental stress, can mediate autoaggregation (Trunk et al., 2018; Kragh et al., 2023). In *P. aeruginosa*, this process can also be triggered by cell-associated polysaccharides (Melaugh et al., 2023), eDNA (Das et al., 2013), and pili (Haussler et al., 2003). Suspended multicellular autoaggregates may furthermore provide protection, initiate colonization, and biofilm formation, and possess similar properties as biofilms, including growth rate, resistance to phagocytes, and drug tolerance (Kragh et al., 2016; Melaugh et al., 2016; Alhede et al., 2011). Additional benefits of close proximity of aggregating cells include improved exchange of signals, genetic material, and co-metabolism. Regulation of autoaggregation may occur through various mechanisms, including changes in transcriptome and proteome, both exoproteome and proteosurfaceome, and post-translational modifications (Afonso et al., 2025). Despite its importance, the QS-dependent mechanisms underlying autoaggregate formation in *P. aeruginosa* remain poorly understood.

Being primarily an extracellular bacterium, *P. aeruginosa* is equipped with a large array of virulence systems contributing to its infection and adaptation. Among them, the QS-regulated secretion systems (SS), namely type 1, 2, 3, 4, 5, and 6 secretion systems (T1SS to T6SS), are ubiquitous and provide translocation of virulent traits, heme-binding proteins, DNA and products for biofilm matrix across the cell envelope. T1SS and T2SS have global functions such as transport and secretion of the extracellular proteome, including an extensive arsenal of metalloproteases, proteases, lipases, phosphatases, and exotoxins. T4SS, unlike the other SS, can transfer DNA in addition to proteins. T3SS and T6SS have specific functions such as the translocation and secretion of toxic effectors into neighboring eukaryotic and prokaryotic cells, and iron transport. Hereby, a large number of cell surface-associated and secreted extracellular proteomes can have a crucial impact on the pathogenesis, iron acquisition, competition, establishment of an infectious niche, colonization, biofilm formation, and horizontal gene transfer-mediated antibiotic resistance (Sauvage and Hardouin, 2020; Pena et al., 2019; Verdial et al., 2023). Proteomic approaches have been applied to gage the molecular basis of biofilm development and the effects of QS in *P. aeruginosa* (Arevalo-Ferro et al., 2003; Hare et al., 2012; Nouwens et al., 2003). Still, a quantitative characterization of exoproteomes is appraised as a challenging but powerful task that should provide important understanding and further insight into bacterial pathogenesis and lifestyle.

This work aimed to assess the pathogenic potential of *P. aeruginosa* by investigating the contribution of the master regulator LasI/RhlI QS system to *P. aeruginosa* community autoaggregation, cell envelope architecture and fitness and extracellular proteome responses, by employing phenotypic assays, nanoscale imaging, and quantitative proteomics. Indeed, in combination with AHL, the LasI/RhlI QS system appears to crucially contribute to pathogenicity potential and accommodation to diverse environments in hosts and habitats.

## Materials and methods

### AHL synthesis

Two AHL, long-chain fatty acid *N*-3-oxo-dodecanoyl-L-homoserine lactone C_16_H_27_NO_4_, MW 297 (3O-C_12_-HSL), and short-chain *N*-butanoyl-L-homoserine lactone, MW 171 (C_4_-HSL), were synthesized by Prof. Peter Konradsson and Lan Bui (Linköping University, Sweden) as previously described (Chhabra et al., 2003). The identity and purity of the synthesized AHL were verified by HPLC, and its activity as a QS-molecule was confirmed by the bioassays described earlier (Winson et al., 1998; Surette and Bassler, 1998). For experiments, AHL compounds were dissolved in 100% dimethylsulfoxide (DMSO) as a stock solution and then further diluted in an aqueous buffer or medium of choice.

### Bacterial growth, treatment with AHL, and preparation of cell culture supernatants (CS)

Four *P. aeruginosa* PA14 strains were used as the model system: the wild type, its single mutants *lasI^−^* and *rhlI^−^*, and double mutant *lasI^−^/rhlI^−^*. The wild type was originally isolated from human burn wounds as UCBPP-PA14 is highly virulent in diverse host models, e.g., in humans, mice, *Caenorhabditis elegans, Drosophila melanogaster*, and *Arabidopsis thaliana* (Rahme et al., 1995; Mikkelsen et al., 2011). The three mutants were constructed by chromosomal deletions in the wild-type PA14 and evaluated by polymerase chain reaction (PCR) (Hoyland-Kroghsbo et al., 2017), and were a kind gift from Prof. Bonnie L. Bassler (Princeton University, NJ). They were routinely cultured in Luria-Bertani (LB) liquid medium or on agar plates overnight at 37 °C. Bacteria were resuspended in a defined minimal medium (Hauser et al., 1998) optimized to produce extracellular proteins, MINS (25 mM KH_2_PO_4_, 95 mM NH_4_Cl, 50 mM C_5_H_8_NO_4_Na monosodium glutamate, 65 mM C_4_H_4_O_4_Na_2_. 6H_2_O disodium succinate, supplemented with freshly made 5 mM MgSO_4_. 7H_2_O, 18 μM FeSO_4_. 7H_2_O, and 2 mM CaCl_2_. 2H_2_O) to A600 of 0.1, further diluted with MINS to initial absorbance of 0.015 at 600 nm (A600). It has been reported that the production of Las and Rhl-controlled proteins (Nouwens et al., 2003; Arevalo-Ferro et al., 2003) and extracellular DNA (Floyd et al., 2016) in *P. aeruginosa* occurs during the exponential and stationary growth phases. For this reason, we chose the 4-h and 18-h cultures grown in MINS (Supplementary Figure S1) for our phenotypic, imaging and proteomic studies. To examine functional complementation, bacteria were treated with a mixture of 5 μM 3O-C_12_-HSL and 5 μM C_4_-HSL and grown for 4 or 18 h at 37° with shaking and aeration. As a vehicle for AHL, DMSO was used. The 18-h bacterial cultures, which reached an A600 of approximatelt1.5, were further processed for autoaggregation assay, transmission electron microscopy (TEM), susceptibility to membrane-damaging agents and antibiotics, and isolation of extracellular protein fraction for proteome analyses. Cell culture supernatants (CS) were recovered after 4-h and 18-h incubations by centrifugation at 5000 g for 20 min at 4 °C, and filtration through 0.45-μm syringe filters (Pall Corporation, Port Washington, NY) to remove bacterial debris. They were further assayed for 3O-C_12_-HSL production, in assays for total protease activity and extracellular DNA (eDNA) release.

### Autoaggregation and sedimentation of bacteria

*Pseudomonas aeruginosa* PA14 wild type and its mutants were grown in MINS for 4 or 18 h at 37° with shaking and good aeration. All growth media were filter-sterilized through 0.45-μm filters (Pall Corporation). Bacterial cells were diluted to A600 of 0.9 in fresh MINS in narrow borosilicate glass tubes. The suspensions were left to autoaggregate and sediment for 5 h at room temperature (RT) by gravity under static conditions. For functional complementation, bacteria were treated with 5 μM 3O-C_12_-HSL and 5 μM C_4_-HSL. To examine the role of proteases and nucleic acids, 15 μM GM6001 (Millipore, Temecula, CA) and 15 U nuclease (Thermo Fisher Scientific, Waltham, MA) were used, respectively. Either inhibitor was added to the cells alone or together with AHL treatment during both growth and setting. DMSO was used as a vehicle. After static incubation, A600 of the top and settled culture fractions were measured with the plate reader SpectraMax iD3 (Molecular Devices, San Jose, CA). Autoaggregation was quantified as a reduction in turbidity at the top of the culture and given as a percentage of the initial A600 values. It was also calculated as the ratio between settled and top culture fractions. The correlation analysis was used to identify and quantify the relationship between autoaggregation ability and total protease activity, as well as autoaggregation ability and eDNA release. The strength of correlation between these variables was evaluated with Pearson’s correlation coefficient. It indicates how closely variables are related, with near −1 or 1 that measures the strength and direction between two variables. Microscopic observation and imaging of autoaggregation in bacterial specimens were performed with a light microscope equipped with a 100 × oil immersion objective. Deconvolution of the images was performed with the Huygens software (Scientific Volume Imaging, Hilversum, The Netherlands). At least four independent experiments in triplicates were done on separate days and with different bacterial passages.

### Quantification of 3O-C_12_-HSL production

The 3O-C_12_-HSL production by PA14 wild type and its three mutants was assessed using the *P. aeruginosa*-derived biosensor strain PA14-R3, a kind gift from Prof. Giordano Rampioni and Prof. Livia Leoni (University Roma Tre, Italy). The PA14-R3 reporter carries a non-functional allele of the *lasI* gene and is thus unable to produce 3O-C_12_-HSL. Still, it harbors the *lasR* gene encoding for the AHL receptor LasR, which senses the exogenous AHL and responds by inducing bioluminescence based on the biosensor strain PA14-R3 (Massai et al., 2011). PA14-R3 was routinely grown on LB agar plates overnight at 37 °C, resuspended in LB MOPS to A600 of 0.18, and further diluted with LB MOPS to A600 of 0.045. Biosensor suspension was aliquoted 195 μL per well in 96-well black plates with clear bottoms. Then, 5 μL of each 4-h CS was added to the wells containing reporter suspension. As controls, DMSO and LB MOPS were used. For the calibration curve, 1:3 serial dilutions of 3O-C_12_-HSL in MINS, with concentrations between 0.005–100 μM, were prepared, and 5 μL of each diluted 3O-C_12_-HSL sample was added to the wells with reporter culture. The plates were incubated for 4 h at 37° with gentle shaking. Bioluminescence and A600 were measured simultaneously with the plate reader SpectraMax iD3. The levels of PA14-R3 activity, which are dependent on the biological activity of AHL, were quantified as the ratio between luminescence and A600. At least four independent experiments in triplicates were done on separate days and with different bacterial passages.

### Total protease activity

The total protease activity was determined according to the manufacturer’s recommendations in 4-h and 18-h CS using the colorimetric protease assay kit 23,263 (Thermo Fisher Scientific), which uses succinylated casein as substrate and trinitrobenzene sulfonic acid (TNBSA). Protease-catalyzed hydrolysis of substrate to peptides yields primary amines that react with TNBSA. This produces an orange-yellow compound with an absorbance intensity at 450 nm (A450), which is proportional to protease activity. Briefly, 50 μL of 4-h or 18-h CS was added to the casein working solution and incubated for 20 min at 37 °C with gentle shaking in the dark. Then, 50 μL of TNBSA working solution was added and incubated further for 20 min at 37 °C. After this, A450 was measured using the plate reader SpectraMax iD3. As the supernatants may give an absorbance background, additional control without the casein substrate was used. The background absorbance values of the substrate-free controls were subtracted from the values obtained for substrate and MINS blank controls to determine the true absorbance intensity due to total protease activity. The level of protease activity was further calculated by normalization of the true absorbance intensity values to the A600 values of the corresponding bacterial culture. At least four independent experiments in triplicates were done on separate days and with different cell passages.

### Extracellular DNA (eDNA) release

The Quant-iT PicoGreen dsDNA reagent (Life Technologies) was used to quantify double-stranded extracellular DNA (eDNA). To obtain extracellular nucleic acid-enriched samples, 4-h and 18-h CS were mixed with 0.1 volume of 3 M sodium acetate, pH 5.2, and 3 volumes of ice-cold 100% ethanol and then precipitated overnight at -20 °C. Pellets were obtained by centrifugation at 13000 g for 30 min at 20 °C, washed twice with 0.5 mL ice-cold 75% ethanol, centrifuged for 10 min each, and then air-dried and resuspended in TE buffer (1 mM EDTA, 10 mM Tris-Cl, pH 8.0). All procedures for the assay were performed according to the manufacturer’s recommendations. Briefly, the working solution of the PicoGreen reagent in TE buffer was mixed, in equal volume, with nucleic acid-enriched sample and incubated for 5 min at 20 °C in the dark. Green fluorescence (excitation 480 nm, emission 520 nm) of the samples was measured with the SpectraMax iD3 plate reader and using TE buffer as a blank control. Fluorescence intensity values were normalized to the A600 values of the corresponding bacterial culture and then extrapolated based on the values obtained for the calibration curve for double-stranded DNA (dsDNA). At least four independent experiments in triplicates were done on separate days and with different bacterial passages.

### Transmission electron microscopy (TEM)

*Pseudomonas aeruginosa* PA14 wild type and its double mutant *lasI^−^/rhlI^−^* were grown in MINS for 18 h at 37 °C with shaking and good aeration. Bacterial cultures were pre-fixed in an equal volume of 6% glutaraldehyde (Polysciences Europe GmbH, Hirschberg an der Bergstrasse, Germany) in 0.1 M Sorensen’s phosphate buffer, pH 7.4 for 5 min at RT, pelleted by centrifugation at 500 g for 10 min at RT, and the pellets were fixed again in 3% glutaraldehyde in 0.1 M Sorensen’s phosphate buffer, pH 7.4 for 2 h at RT. Cells were harvested by centrifugation at 500 g for 15 min at RT, the supernatant was discarded and the pellet was washed with the same buffer and post-fixed in 1% osmium tetroxide (Polysciences Europe GmbH) for 1 h at 4 °C. After two washing steps in the same phosphate buffer, a dehydration step with 50% ethanol and an *en bloc* staining with 2% uranyl acetate (Polysciences Europe GmbH) in 70% ethanol were applied. Dehydration was continued with a series of ascending concentrations of ethanol (90%, 2× 100%) and two steps in anhydrous acetone. Infiltration in epoxy embedding medium, first in three mixtures of acetone-embedding medium (3:1, 1:1, 1:3) and then in 100% embedding medium, was performed before embedding (48 h at 60 °C) in Araldite/Embed 812 epoxy embedding medium (Electron Microscopy Sciences, Hatfield, PA). Ultrathin sections of 70-nm thickness were prepared using a Leica EM UC7 ultramicrotome (Leica Microsystems GmbH, Vienna, Austria), collected onto formvar-coated slot grids, and counter-stained with uranyl acetate and lead citrate (Polysciences Europe GmbH). High-resolution images were taken using a JEM 1400 Flash transmission electron microscope (JEOL Ltd., Tokyo, Japan) operated at 80 kV and equipped with a XAROSA camera and RADIUS software (EMSIS GmbH, Munster, Germany). The cell envelope curvature in the TEM images was quantified, since it can serve as an indicator of membrane responses and, potentially, drug susceptibility. The average radius of the cell envelope curvature and the number of the cell surface-associated biopolymeric components were measured and quantified for a minimum of 50 cells for each condition using the ImageJ Fiji software (NIH, Bethesda, MD). The experiments were done 3 times on separate days and with different cell passages.

### Susceptibility to membrane-damaging agents

Tolerance to membrane permeabilizing and damaging solvents, sodium dodecyl sulfate (SDS, lipid removing anionic surfactant) and ethylenediaminetetraacetic acid (EDTA, Ca^2+^, and LPS disturbing agent) was tested. *P. aeruginosa* PA14 wild type and its mutants were grown in MINS for 18 h at 37 °C with shaking and aeration, reaching A600 of approximately 1.5. Bacterial cultures were diluted to A600 of 0.1 in fresh MINS supplemented with either 0.25% SDS, 1 mM EDTA or both compounds and incubated for 2.5 h at 37 °C with agitation and aeration. Suitable dilutions of bacterial suspensions were spread on LB agar plates and incubated for 18–24 h at 37 °C. Cell survival was quantified, and the number of viable colony forming units per ml (CFU/ml) was normalized against control, wild-type PA14 cells treated with diluent (100%). At least four independent experiments were done on separate days and with different bacterial passages.

### Susceptibility to antibiotics

Antimicrobial susceptibility to a range of antibiotics was tested by determination of minimal inhibitory concentration (MIC) using the broth microdilution method. Sensititre gram-negative GN2F panel in 96-well plates (Thermo Fisher Scientific) included 23 antibiotics of several classes, including front-line antipseudomonal drugs, e.g., piperacillin/tazobactam, ceftazidime, imipenem, meropenem, ciprofloxacin, tobramycin, and amikacin. All procedures for the method were performed according to the manufacturer’s recommendations. Briefly, *P. aeruginosa* PA14 wild-type and its mutants were grown in Mueller–Hinton (MH) broth overnight at 37 °C with shaking and aeration. Then, 50 μL of bacterial suspension diluted to 5 × 10^5^ CFU/mL in MH broth was added to the wells containing the antibiotic dilutions. The plates were further incubated for 20 h at 37 °C and evaluated by visual inspection. MIC was defined as the lowest concentration of the drug (in μg/ml) that prevented visual growth. MIC values 2-fold or greater than that of the control, wild-type PA14 cells treated with diluent, were considered significant. The experiments were done 3 times on separate days and with different cell passages.

### Isolation of extracellular proteins

*Pseudomonas aeruginosa* PA14 wild-type and its double mutant *lasI^−^/rhl^−^* were grown in MINS for 18 h at 37 °C with shaking and good aeration, reaching A600 of approximately 1.5 in the late exponential phase—early stationary phase. Then, a Halt Protease Inhibitor Cocktail (Thermo Fisher Scientific) was added to bacterial cultures. Cells were removed by centrifugation at 5000 x g for 15 min at 4 °C, and supernatants were filtered using 0.45-μm syringe filters (Pall Corporation) to remove bacterial debris. Cell-free supernatants were concentrated using 3 K MWCO 20 mL devices (Pall Corporation) and centrifugation at 5000 g for 30 min at 17 °C. Concentrated samples were washed and desalted using the same devices by adding 10 mL 50 mM ammonium bicarbonate and centrifugation at 5000 g for 20 min at 17 °C; this step was repeated thrice. Finally, the samples were concentrated at 5000 g for 1 h at 17 °C, resulting in a final volume of approximately 1 mL extracellular protein fraction.

### In-solution digestion

The extracellular protein fractions were reduced in 5 mM DTT for 30 min at 60 °C and alkylated with 20 mM IAA for 30 min at RT in the dark. DTT was then added again to the samples to a final concentration of 17.5 mM. MS-grade trypsin (Thermo Fisher Scientific) was used for the following enzymatic digestion according to the manufacturer’s recommendations. Pierce C18 tips (Thermo Fisher Scientific) were used to desalt the obtained peptides. These were then reconstituted in 0.1% formic acid in milliQ water, and peptide concentrations were estimated at A280 (NanoDrop ND-1000 Spectrophotometer, Thermo Fisher Scientific) before liquid chromatography–tandem mass spectrometry (LC–MS/MS) analyses.

### Proteomic analysis with LC–MS/MS

Peptides were separated by reverse phase chromatography on a 20 mm × 100 μm C18 pre-column followed by a 100 mm × 75 μm C18 column with particle size 5 μm (NanoSeparations, Nieuwkoop, The Netherlands) at a flow rate of 300 nL/min on EASY-nLC II (Thermo Fisher Scientific) by a gradient of 0.1% formic acid in water (A) and 0.1% formic acid in acetonitrile (B) as follows: from 2% B to 30% B in 60 min; from 30% B to 100% B in 60 min. Automated online analyses were performed in positive mode with LTQ Orbitrap Velos Pro hybrid mass spectrometer (Thermo Fisher Scientific) equipped with a nano-electrospray source with Xcalibur software version 2.6 (Thermo Fisher Scientific). Full MS scans were collected with a range of 350–1800 m/z at a resolution of 30,000 (m/z 200), the top 20 most intense multiple charged ions were selected with an isolation window of 2.0 and fragmented in the linear ion trap by collision-induced dissociation with a normalized collision energy of 30%. Dynamic exclusion for 60 s was enabled, ensuring that peaks selected for fragmentation were excluded.

### Proteome database search

Generated raw files were analyzed with Sequest HT in Proteome Discoverer CS version 1.4.0.288 (Thermo Fisher Scientific) using the strain-specific *P. aeruginosa* PA14 protein sequence database available at NCBI^1^. Proteins were identified with the following search parameters: trypsin as a digestion enzyme; maximum number of missed cleavages 2; fragment ion mass tolerance 0.50 Da; parent ion mass tolerance 10.0 ppm; carbamidomethylation of cysteine as a fixed modification and methionine oxidation as a variable modification.

### Proteomic data evaluation and label-free quantification

Identified proteins were validated using the SCAFFOLD software version 4.4.8 (Proteome Software Inc., Portland, OR). Identifications were based on a minimum of two peptides, a minimum of 95% peptide identification probability (using the Scaffold Local FDR algorithm), and a minimum of 99% protein identification probability using the Protein Prophet algorithm (Nesvizhskii et al., 2003). Proteins containing similar peptides that could not be differentiated based on LC–MS/MS analysis alone were grouped to satisfy the principles of parsimony. The label-free quantitative analysis was done using a total number of spectral counts; normalization was performed to account for variations between samples. Quantitative differences were statistically analyzed by ANOVA test using SCAFFOLD software. Additionally, quantitative differences for LasB were also statistically analyzed by Student’s *t*-test using SCAFFOLD software. Differences with *p*-values ≤ 0.05 were considered statistically significant.

### Protein homology search

Protein sequences were annotated by search in the NCBI database using the Basic Local Alignment Search Tool (BLAST, National Center for Biotechnology Information, Bethesda, MD) blast-p algorithm and Uniprot BLAST (EMBI-EBI, Cambridgeshire, UK) software^2^.

### Statistical analyses

Data in the bar graphs are presented as mean ± SE, and statistical analyses are based on a two-tailed Student’s *t*-test. *p*-values < 0.05, < 0.01, and < 0.001 were considered significant. The strength of correlation between variables was evaluated with Pearson’s correlation coefficient. This is also specified in the figure legends. For the autoaggregation assay, quantification of 3O-C_12_-HSL, total protease activity, and production of extracellular DNA, at least four independent experiments in triplicates were done on separate days on different bacterial passages. The TEM experiments were done three times on separate days and with different cell passages. The experiments for the proteome were repeated at least 4–6 times on separate days, and data evaluation, quantification, and statistical analyses are described above.

## Results

### More effective autoaggregation of wild-type *Pseudomonas aeruginosa* PA14 than *lasI^−^/rhlI^−^* mutant

**Figure 1:**
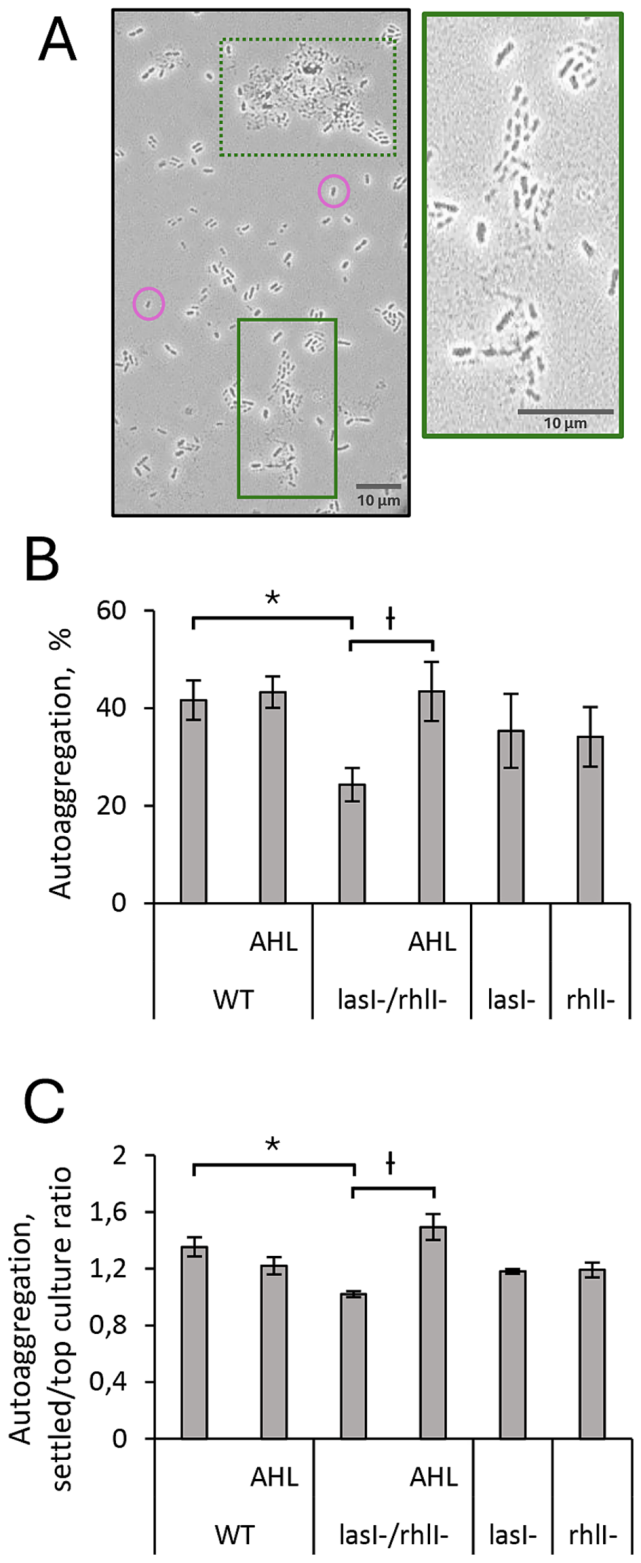
Autoaggregation of *P. aeruginosa* PA14 wild-type and its *lasI-/rhlI-* mutants. Wild-type bacteria (WT), *lasI-*, *rhlI-* and *lasI−/rhl-* mutants were treated with DMSO as a diluent control. For functional complementation, bacteria were treated with a mixture of 5 μM 3O-C_12_-HSL and 5 μM C_4_-HSL (AHL). The bacterial suspensions were assessed for autoaggregation and sedimentation under static conditions. **(A)** Microscopic imaging of autoaggregation of the representative bacterial specimens. Image regions within solid and dashed green colored boards highlight bacterial aggregates. Non-aggregated cells are shown in pink circles. The image insert with a solid green board represents a zoomed view of the cell aggregate at higher magnification. Scale bars represent 10 μm. **(B)** Quantification of autoaggregation was calculated as a reduction in turbidity at the top of the culture and presented as a percentage of the initial A600 values. **(C)** Quantification of autoaggregation is presented as the ratio between settled and top culture fractions. Columns represent the means ± SE. Significant differences in comparison to WT are indicated with * when *p* < 0.05, as analyzed by two-tailed paired Student’s *t*-test. Significant differences in comparison to *lasI−/rhl-* are indicated with Ɨ when *p* < 0.05 as analyzed by two-tailed Student’s *t*-test. Data from 4 independent experiments performed on separate days and with different cell passages.

We explored the formation of suspended cellular aggregates in wild-type *P. aeruginosa* in comparison to its *lasI^−^, rhlI^−^* and *lasI^−^/rhlI^−^* mutants. After a 5-h settling and further light microscopic analysis, we assessed the formation of cellular aggregates yielding approximately 10–60 μm in size within the planktonic cultures (Figure 1A). Quantitatively, wild-type bacteria displayed a significant, 1.7-fold greater autoaggregation ability compared to the QS defective *lasI^−^/rhlI^−^* mutant. The levels of aggregation activity in *lasI^−^* and *rhlI^−^* mutants were slightly but not significantly decreased compared to wild-type bacteria (Figures 1B,C). We predicted that the accumulation of AHL, which occurs naturally in bacteria during growth and transition to exponential and stationary phases, should trigger cellular aggregation. We thus next determined whether autoaggregation ability could be functionally complemented by pre-treatment of the double mutant with a mixture of synthetic 5 μM 3O-C_12_-HSL and 5 μM C_4_-HSL. Indeed, it appeared that AHL was able to significantly elevate the formation of aggregates in *lasI^−^/rhlI^−^* mutant to a level similar to the wild type (Figures 1B,C). Taken together, this suggests that wild-type *P. aeruginosa* is more prone to form cellular aggregates compared to the strains with deletions of the *lasI* and *rhlI* genes; exogenous supplementation of AHL complemented the aggregation, which points to the QS control of the process.

### Impact of protease activity on the formation of cellular aggregates in *Pseudomonas aeruginosa* PA14

**Figure 2:**
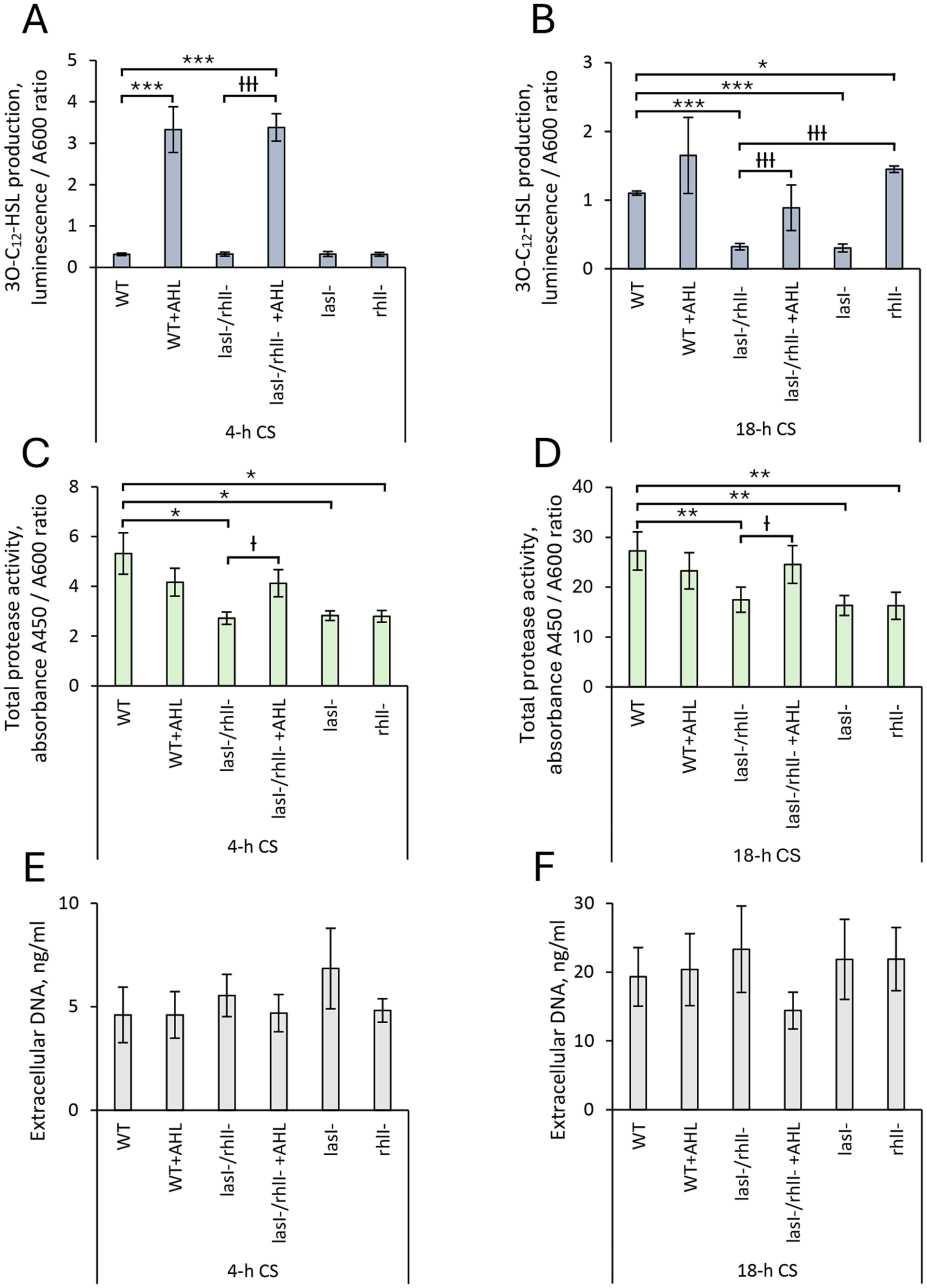
Evaluation of phenotypic characteristics in *P. aeruginosa* PA14 wild-type and *lasI-/rhlI-* mutants. Wild-type bacteria (WT), *lasI-*, *rhlI-* and *lasI−/rhl-* mutants were treated with DMSO as a diluent control. For functional complementation, bacteria were treated with a mixture of 5 μM 3O-C_12_-HSL and 5 μM C_4_-HSL (AHL). Cell culture supernatants (CS) were recovered after 4-h growth (4-h CS) and 18-h growth (18-h CS) and proceeded for phenotypic assays. **(A)** Analysis of 3O-C_12_-HSL in 4-h CS and **(B)** 18-h CS using reporter PA14-R3. The levels of 3O-C_12_-HSL production were quantified as the ratio between luminescence and A600. **(C)** Total protease activity in 4-h CS and **(D)** 18-h CS was measured using the colorimetric protease assay and quantified by normalization of the A450 values to the A600 values of the corresponding bacterial culture. **(E)** Extracellular DNA release in 4-h CS and **(F)** 18-h CS was measured by Quant-iT PicoGreen dsDNA reagent. Fluorescence intensity values were normalized to the A600 values of the corresponding bacterial culture and extrapolated based on the values obtained for the calibration curve for dsDNA. At least 4 independent experiments in triplicates were conducted on separate days and with different bacterial passages. Columns represent the means ± SE. Significant differences in comparison to WT are indicated with * when *p* < 0.05, ** when *p* < 0.01 and *** when *p* < 0.001 as analyzed by two-tailed Student’s *t*-test. Significant differences in comparison to *lasI−/rhl-* are indicated with Ɨ when *p* < 0.05 and ƗƗ when *p* < 0.01 and ƗƗƗ when *p* < 0.001, as analyzed by two-tailed Student’s *t*-test.

Molecules of several different types, including extracellular compounds, may act as autoagglutinins and thereby mediate the formation of bacterial aggregates through extracellular interactions (Kragh et al., 2023). For this reason, we first measured comparatively the production of 3O-C_12_-HSL, total protease activity and extracellular DNA (eDNA) release in 4- and 18-h cell culture supernatants (CS) isolated from wild-type *P. aeruginosa,* its QS mutants, as well as the response to AHL. As expected, after 18 h, but not 4 h, 3O-C_12_-HSL production in wild-type bacteria and *rhl^−^* mutant was significantly, about 3-fold higher than in the *lasI^−^* and *lasI^−^/rhlI^−^* mutants (Figures 2A,B). Exogenous supplementation of AHL increased the 3O-C_12_-HSL production and abolished the difference observed between the wild type and double mutant (Figure 2B). Furthermore, we detected that wild-type *P. aeruginosa* displays significantly, between 1.5- and 1.9-fold greater protease activity in contrast to the *lasI^−^, rhlI^−^* and *lasI^−^/rhlI^−^* mutants. Stimulation with AHL eliminated the difference seen in the total protease activity between the wild type and the double mutant (Figures 2C,D). The pattern of eDNA release appeared similar in wild-type *P. aeruginosa*, its QS mutants, and also when the AHL mixture was added (Figures 2E,F).

**Figure 3:**
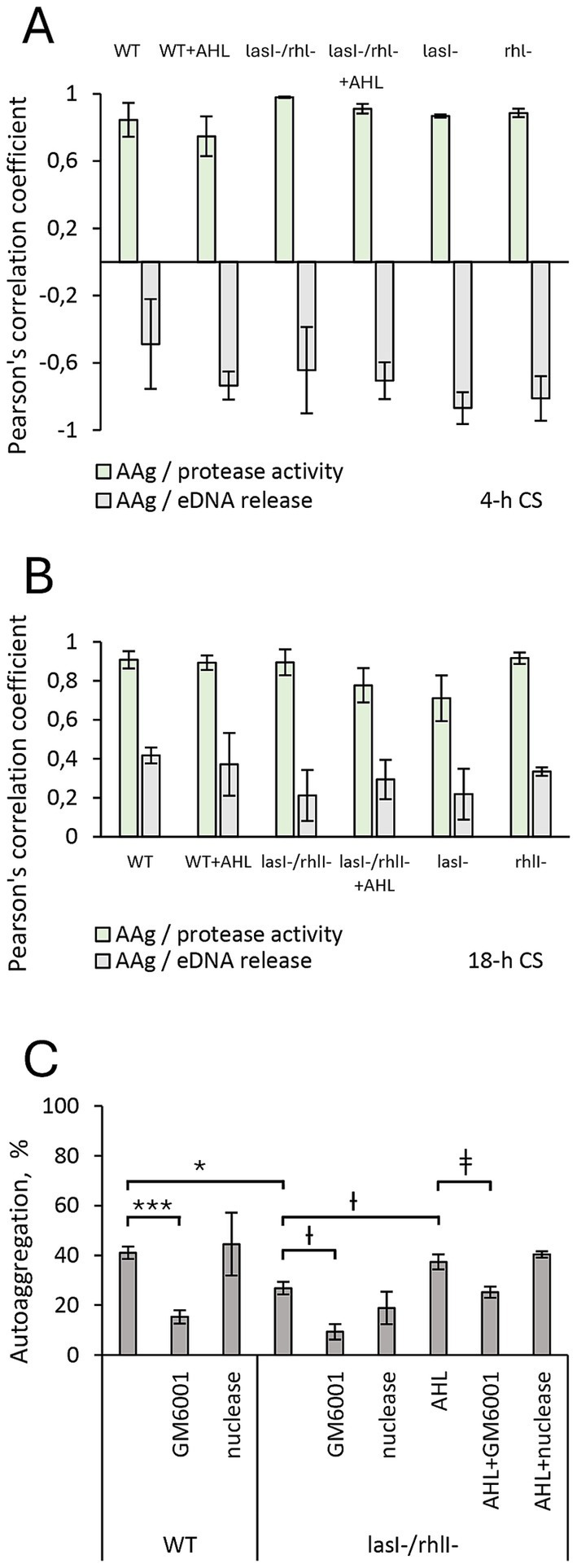
Relationship between autoaggregation, protease activity, and eDNA release in *P. aeruginosa* PA14. Wild-type bacteria (WT), *lasI-, rhlI-* and *lasI−/rhl-* mutants were treated with DMSO as a diluent control. For functional complementation, bacteria were treated with a mixture of 5 μM 3O-C12-HSL and 5 μM C4-HSL (AHL). Cell culture supernatants (CS) were recovered after 4-h growth (4-h CS) and 18-h growth (18-h CS). Correlation between autoaggregation (AAg) and protease activity (green columns), and between autoaggregation and eDNA release (gray columns) in **(A)** 4-h CS and **(B)** 8-h CS is presented as Pearson’s coefficient values. **(C)** The impact of proteases and eDNA on the autoaggregation in wild-type *P. aeruginosa*, *lasI-/rhlI-* mutant, and in response to AHL. The bacterial suspensions were assessed for autoaggregation and sedimentation under static conditions. Either 15 μM GM6001 protease inhibitor or 15 U nuclease was added to the cells alone or together with AHL treatment during growth and setting. Quantification of autoaggregation was calculated as a reduction in turbidity at the top of the culture and given as a percentage of the initial A600 values. At least 4 independent experiments in triplicates were conducted on separate days and with different bacterial passages. Columns represent the means ± SE. Significant differences in comparison to WT are indicated with * when *p* < 0.05, ** when *p* < 0.01 and *** when *p* < 0.001 as analyzed by two-tailed Student’s *t*-test. Significant differences in comparison to *lasI−/rhl-* are indicated with Ɨ when *p* < 0.05. ǂ Significant differences in comparison to *lasI−/rhl-* + AHL are indicated with ǂ when *p* < 0.05.

Next, using statistical tools, we identified and quantified how changes in autoaggregation are associated with protease activity and eDNA release in *P. aeruginosa* wild-type and its mutants, as well as in response to AHL, assuming that this analysis would provide further insights into the topic. Accordingly, the correlation between autoaggregation and either protease activity or eDNA release was calculated for the 4-h (Figure 3A) and 18-h time points (Figure 3B). Pearson’s coefficient values between 0.7 and 1 were obtained for autoaggregation and protease activity, which indicates a good correlation. For autoaggregation and eDNA, the values varied between −0.9 and 0.4, which points to no or low correlation (Figures 3A,B).

The observed pattern in the correlation analysis led us to further explore the impact of proteases and eDNA on the bacterial autoaggregation. Here, treatment with the protease inhibitor GM6001 resulted in significant, 2.6- and 2.8-fold decreased autoaggregation in the wild-type bacteria and double mutant, respectively (Figure 3C). The addition of AHL mixture alone or together with the protease inhibitor GM6001 elevated and restored the level of aggregate formation in the double mutant. Supplementation of nuclease did not affect the autoaggregation ability of either the wild-type bacteria or the double mutant (Figure 3C).

These results suggest that functional AHL-based QS circuits and protease activity act synergistically on the suspended aggregation in *P. aeruginosa* PA14.

### Ultrastructure of the cell envelope in *Pseudomonas aeruginosa* PA14 wild type and *lasI^−^/rhlI^−^* mutant

**Figure 4:**
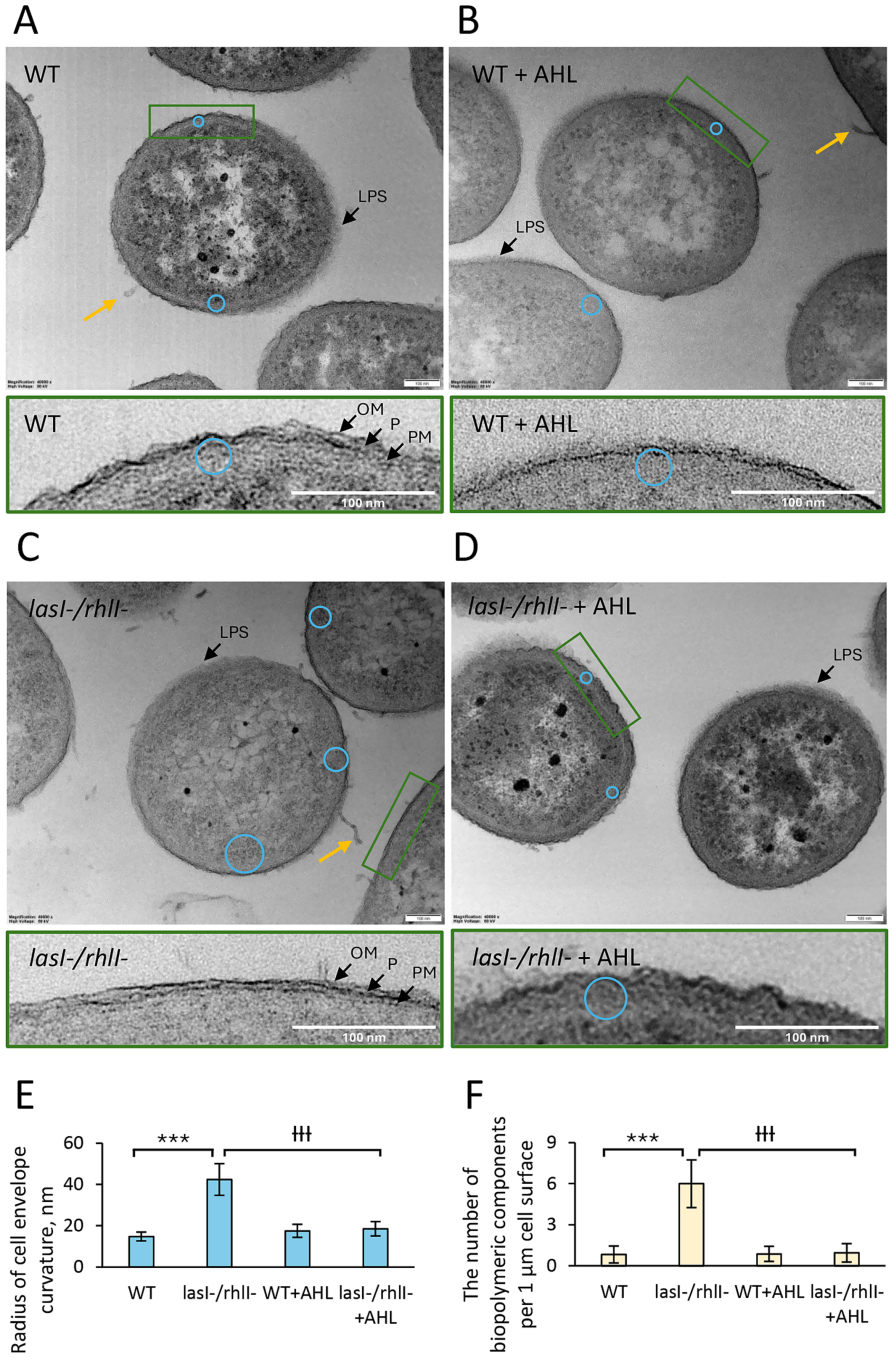
High-resolution visualization of the nanostructure of *P. aeruginosa* PA14 wild-type and *lasI-/rhlI-* mutant. **(A)** Wild-type bacteria (WT) were treated with DMSO as a diluent control or **(B)** exposed to a mixture of AHL for 18 h (WT + AHL). **(C)** The double mutant (*lasI-/rhlI-*) was treated with DMSO as a diluent control or **(D)** exposed to a mixture of AHL for 18 h (*lasI-/rhlI-* + AHL). Cells were fixed, stained with uranyl acetate, and analyzed by TEM. The radius of the cell envelope curvature is shown as blue circles. Inserts with green boards represent zoomed views of the cell envelope at higher magnification. The cytoplasm and cytoplasmic components, including nucleoid in bacteria, can be distinguished, respectively, as less electron-dense (lighter) and comparatively higher electron-dense (darker) regions. Bacterial cells clearly show plasma membrane (PM), cell wall and periplasmic space (P), outer membrane (OM), and lipopolysaccharide (LPS) around the bacteria (black arrows). Additional structures were observed between cells and associated with cell surfaces that visually resemble extracellular biopolymers or cell debris (yellow arrows). Scale bars represent 100 nm. **(E)** Quantification of cell envelope curvature and **(F)** the number of cell surface-associated biopolymeric components. The means ± SE are based on the data from 3 independent experiments and a minimum of 50 cells for each condition. *p*-values < 0.05 (*), < 0.01 (**), and < 0.001 (***) were considered significant in comparison to WT; *p*-values < 0.05 (Ɨ), < 0.01 (ƗƗ), and < 0.001 (ƗƗƗ) were considered significant in comparison to *lasI-/rhlI-*, as calculated by two-tailed paired Student’s *t*-test.

Las/Rhl QS system controls the production of a large array of cell surface-associated and secreted proteins, which may modify cell surface architecture and fitness. These features may also have important consequences for the behavior of the bacterial populations, including variations in membrane functions (Lapinska et al., 2022). Therefore, we employed TEM to reveal and analyze the ultrastructure of wild-type *P. aeruginosa* PA14 and *lasI^−^/rhlI^−^* mutant. Both strains were treated with DMSO as a diluent control (Figures 4A,C) or exposed to a mixture of 5 μM 3O-C_12_-HSL and 5 μM C_4_-HSL for 18 h (Figures 4B,D). The intracellular morphology of the cytoplasm and cytoplasmic components, together with the nucleoid, was similar across all groups. Most notably, we revealed the alterations in the ultrastructure of the cell envelope, including its morphology and cell surface-associated polymeric components (Figure 4). We predicted that cell envelope nanoarchitecture and outline can serve as an indicator of cell envelope fitness. In wild-type bacteria treated with either diluent or AHL mixture, cell envelopes appeared well-structured and displayed all layers: LPS-enriched outer membrane (OM), cell wall with periplasmic space (P) and plasma membrane (PM) (Figures 4A,B). The wild-type bacteria also exhibited pronounced and regular undulations of the cell envelope, indicated by shaping into a series of ridges and grooves with a small radius of curvature, about 15–20 nm (Figures 4A,B,E). The number of polymeric components associated with the cell surface was low (Figure 4F). The double mutant treated with diluent still maintained the typical structural layers of the cell envelope (Figure 4C). However, it exhibited subtle undulations in the cell envelope, as indicated by a larger radius of curvature, around 40 nm (Figures 4C,E). The number of polymeric components associated with the cell surface was elevated (Figures 4C,F). We also observed that treatment of the double mutant with AHL mixture for 18 h resulted in prominent undulations of the cell envelopes, resembling those of the wild type (Figures 4D,E). Consistent with this, the number of polymeric components associated with the cell surface was low (Figures 4D,F). Together, these high-resolution imaging data demonstrate that the ultrastructure of the cell envelope in *P. aeruginosa* PA14 is also dependent on an intact LasI/RhlI system.

### Tolerance to membrane-damaging agents and antibiotics in *Pseudomonas aeruginosa* PA14

**Figure 5:**
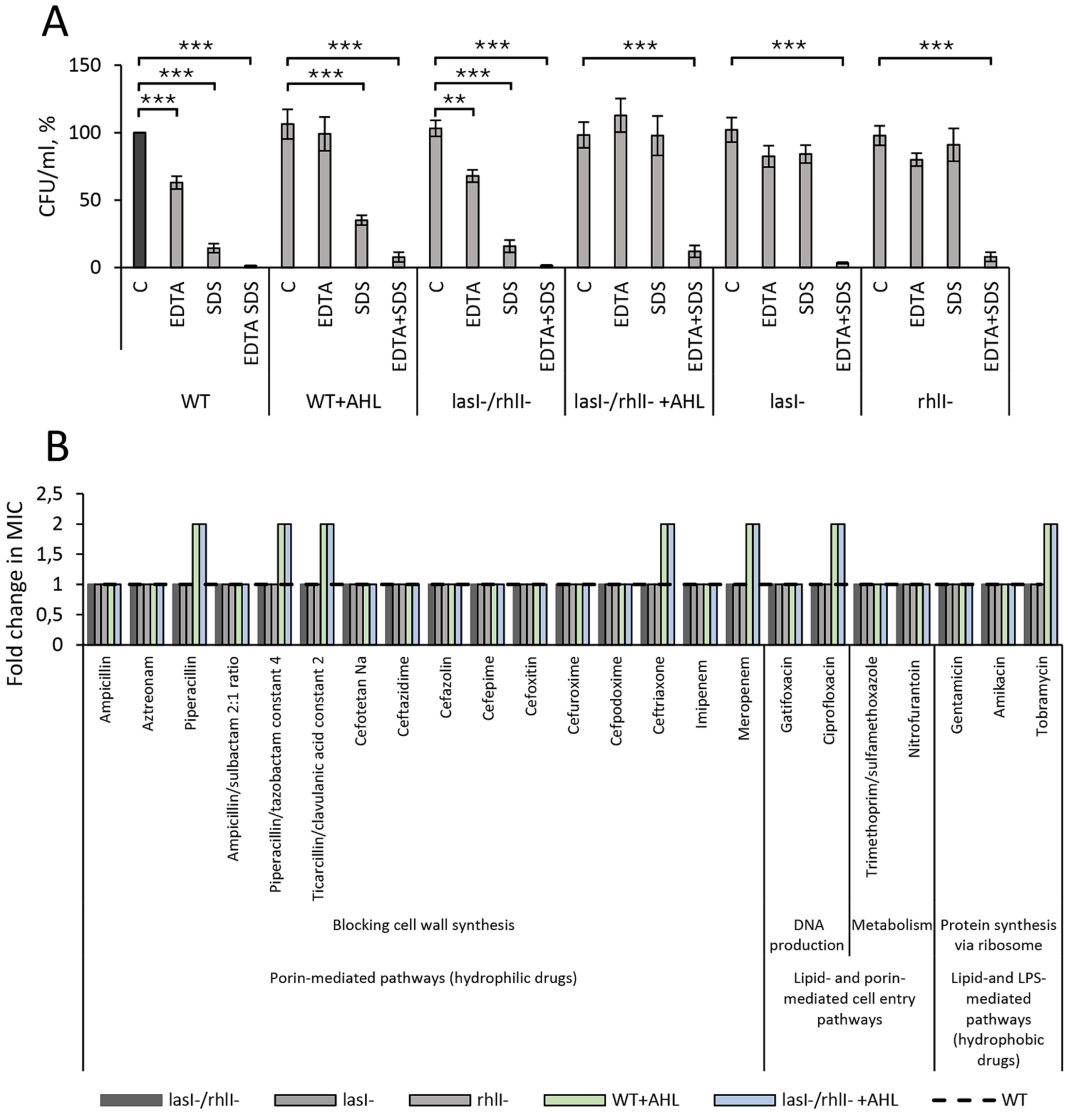
Phenotypic changes in *P. aeruginosa* PA14 wild-type, *lasI-/rhlI-* mutant and in response to AHL indicate alterations in its outer membrane fitness. **(A)** Susceptibility to membrane-damaging agents, EDTA, and SDS. Wild-type bacteria (WT), mutants *lasI-* and *rhlI-,* and double mutant (*lasI-/rhlI-*) were treated with DMSO as a diluent control (C) or a mixture of AHL for 18 h. Bacterial cultures were exposed to SDS, EDTA, or both compounds (EDTA+SDS) for 2.5 h. Bacterial growth was quantified by the number of viable colony-forming units per milliliter (CFU/ml) and normalized against wild-type bacteria treated with diluent (WT C, black bar), which is set as the 100% reference baseline. The bars represent CFU/ml in %, displaying the mean ± SE, based on data from at least 4 independent experiments performed on separate days and with different cell passages. Significant differences were analyzed by two-tailed Student’s *t*-test for each condition in comparison to C of the corresponding strain and conditions and are indicated with ** when *p* < 0.01 and *** when *p* < 0.001. **(B)** Relative change of minimum inhibitory concentration (MIC) for antibiotics of different groups according to their mechanism of action and cell entry pathways. Wild-type bacteria (WT), mutants *lasI-* and *rhlI-*, and double mutant (*lasI-/rhlI-*) were treated with DMSO as a diluent control or a mixture of AHL for 18 h. MIC was determined by the broth microdilution method and confirmed by 3 independent experiments conducted on separate days and with different bacterial passages. Fold changes were quantified with respect to WT, represented as a dotted black line. MIC differences of at least 2-fold in comparison to WT were considered significant.

There is growing evidence that the complexity, dynamics, and remodeling of bacterial membranes can influence drug tolerance (Lapinska et al., 2022; Lithgow et al., 2023; Larrouy-Maumus et al., 2016). The observed ultrastructural features of the cell envelope, which were dependent on an intact LasI/RhlI system, led us to investigate bacterial tolerance to membrane-damaging agents and antibiotics. Wild-type *P. aeruginosa* and the *lasI^−^/rhlI^−^* mutant were susceptible to the action of EDTA (Ca^2+^ and LPS disturbing agent), SDS (lipid removing anionic surfactant) or the combination of both agents (membrane permeabilization). Treatment with these membrane-damaging agents resulted in similar viability reduction in both the double mutant and the wild-type bacteria. The single *lasI^−^* and *rhlI^−^* mutants were more tolerant to EDTA and SDS. Still, supplementation with the AHL mixture led to more efficient survival in either bacterium (Figure 5A), providing partial tolerance to these membrane-damaging agents. In line with this, the patterns of antimicrobial susceptibility in wild-type bacteria and *lasI^−^, rhlI^−^* and *lasI^−^/rhlI^−^* mutants were similar. The addition of AHL mixture to either wild-type bacteria or *lasI^−^/rhlI^−^* mutant resulted in elevated resistance to the following drugs: some *β*-lactam antibiotics that use porin-mediated uptake, e.g., piperacillin, ticarcillin, ceftriaxone and meropenem; the fluoroquinolone agent ciprofloxacin that is taken up via lipid- and porin-mediated pathways; and the aminoglycoside agent tobramycin that uses lipid- and LPS-mediated uptake for entry (Figure 5B). Together, these data demonstrate that AHL-based QS signaling may provide *P. aeruginosa* with a partial tolerance to membrane-damaging and antimicrobial agents.

### Alterations in the extracellular proteome in *Pseudomonas aeruginosa* PA14 wild-type and *lasI^−^/rhlI^−^* mutant

**Figure 6:**
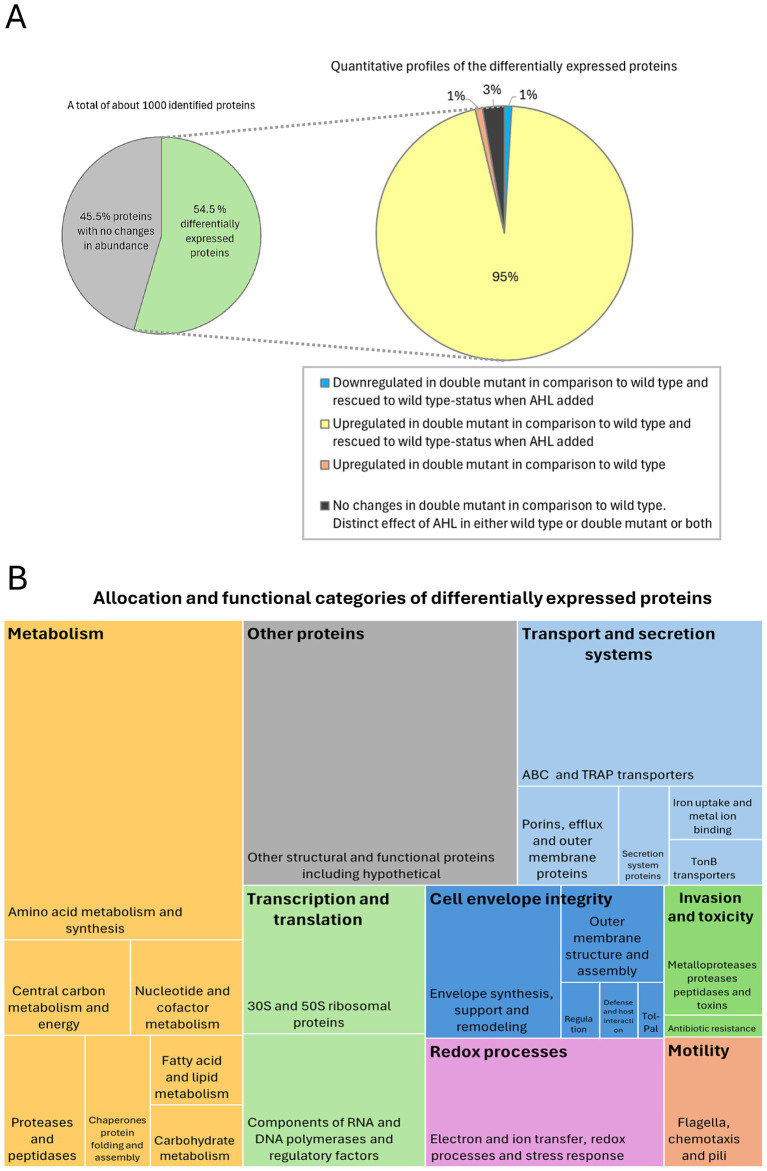
Summary of the alterations in the extracellular proteome in *P. aeruginosa* PA14 wild-type and *lasI-/rhlI-* mutant. LC–MS/MS was employed to quantitatively analyze the extracellular proteomes of wild-type *P. aeruginosa* and its *lasI-/rhlI-* mutant, where both were exposed to a mixture of AHL for 18 h, or treated with DMSO as a diluent control. **(A)** Multi-level statistical pie chart visualization of the quantitative profiles of the extracellular proteome. A circular graph on the left illustrates about 45.5% of proteins with no changes (gray sector) and about 54.5% differentially expressed proteins (green sector) among a total of about 1,000 identified proteins and protein clusters. A circular graph on the right displays the details for the quantitative profiles of differentially expressed proteins. According to ANOVA tests, only about 1% proteins were significantly downregulated in the double mutant in comparison to the wild type and rescued to wild type-status when AHL was added (blue sector). Most notably, about 95% of differentially expressed proteins were upregulated in the double mutant in comparison to the wild type and returned to wild type-status when the AHL mixture was added (yellow sector). About 1% of proteins were upregulated in the double mutant in comparison to the wild type (orange sector). Only about 3% of proteins were not significantly changed in the extracellular space of the double mutant in comparison to wild-type bacteria (black sector). **(B)** The tree map visualization of the allocation and functional categories of differentially expressed proteins. Based on NCBI GO analyses, we could allocate differentially expressed proteins to the major functional groups shown as boxes in different colors and the corresponding names of the functional groups, which are depicted in black bold text in the upper part of the box. The largest boxes consist of several rectangles of the same color, which represent functional undergroups of proteins. The sizes of each box and rectangle are proportional to the number of identified proteins.

To advance our knowledge on bacterial virulence, adaptation and interactions with the host, we further addressed the molecular mechanisms and patterns involved in QS-controlled secreted proteome in *P. aeruginosa*, and particularly the impact of the tandem AHL-dependent LasI/RhlI system. Therefore, we used LC–MS/MS to quantitatively analyze the extracellular proteomes of wild-type *P. aeruginosa* and its *lasI^−^/rhlI^−^* mutant. Both were exposed to a mixture of 5 μM 3O-C_12_-HSL and 5 μM C_4_-HSL for 18 h or treated with DMSO as a diluent control. The bacteria were harvested, and extracellular protein fractions were obtained in the late exponential and early stationary phase, when most QS-regulated proteins are supposed to be (Arevalo-Ferro et al., 2003; Sauvage and Hardouin, 2020; Scott et al., 2013). The majority of about 1,000 identified proteins and protein clusters were classified as extracellular according to NCBI GO annotations. These proteins were associated with the outer membrane, periplasm, plasma membrane, cytoplasm, flagellum and secretion machinery (Figures 6A,B; Supplementary Tables S1–S6). Exoproteins, e.g., the metalloproteases AprA, ImpA and LasA, were detected as the most abundant secreted proteins in all groups of samples. It should also be noted that many identified proteins were annotated as hypothetical with uncertain localization and function. Indeed, about 40% of the *P. aeruginosa* proteins are annotated as hypothetical or not yet studied (Armengaud et al., 2013; Ball et al., 2016). It can also be mentioned that some typically cytosolic proteins can be released through the TSS and outer membrane vesicles, acting as a cargo for intercellular delivery and signaling. In addition, some of them might have undiscovered functional roles outside the cell, contributing to the bacterial community or their interactions with the environment (Caruana and Walper, 2020). Lysis artifacts are less likely according to our imaging data from TEM (Figure 4). The MW of the proteins varied between 5 and 203 kDa, where 79% of them were below 50 kDa and about 20% were even smaller, under 25 kDa.

Further, we identified a total of 545 proteins (about 54.5%) that were differentially expressed (Figure 6A) when we compared the four groups using ANOVA: (1) wild-type bacteria treated with DMSO as a diluent control; (2) *lasI^−^/rhlI^−^* mutant treated with DMSO; (3) wild-type bacteria exposed to 5 μM 3O-C_12_-HSL and 5 μM C_4_-HSL for 18 h; and (4) *lasI^−^/rhlI^−^* mutant exposed to 5 μM 3O-C_12_-HSL and 5 μM C_4_-HSL for 18 h. Based on NCBI GO analyses, we allocated 443 of 545 differentially expressed proteins to the following seven major functional groups: metabolism; transcription and translation; transport and secretion systems; cell envelope integrity; redox processes; invasiveness and toxicity; and motility (Figure 6B; Supplementary Tables S1–S6). The remaining 102 differentially expressed proteins were either allocated to other functional groups or annotated as hypothetical (Figure 6B).

Four proteins associated with enzymatic proteolysis of host connective tissues and hereby invasiveness, namely the metalloproteases AprA, ImpA, and LasA, and alkaline protease secretion protein AprF, were significantly downregulated in the double mutant in comparison to wild type and rescued to wild type-status when AHL was added (Figures 6A,B; Supplementary Table S6).

Most notably, according to the ANOVA analysis, about 500 proteins (about 95% of those differentially expressed) were upregulated in the double mutant in comparison to the wild type and returned to wild-type status when the AHL mixture was added (Figure 6A). Here, the largest group of about 180 proteins included those involved in the cellular metabolism of amino acids, carbon, carbohydrates, nucleotides and cofactors, proteases and peptidases, fatty acids and lipids, chaperones, protein folding and assembly (Figure 6B; Supplementary Table S1). A set of proteins involved in transcription and translation was represented by 38 structural ribosomal proteins that build up the 30S and 50S subunits, as well as 34 components of RNA and RNA polymerases, and regulatory proteins and factors (Figure 6B; Supplementary Table S2). Yet another large group of about 90 proteins being crucial for transport and secretion systems comprised ABC, TRAP and TonB transporters, porins, efflux and outer membrane proteins, secretion system, and iron uptake networks (Figure 6; Supplementary Table S3). A large assembly of proteins that stand for cell envelope integrity was involved in cell envelope synthesis, support, remodeling and degradation; outer membrane structure and assembly; envelope regulation and adaptation; defense and host interaction; and specifically, the Tol-Pal system for envelope maintenance (Figure 6B; Supplementary Table S4). Another group of approximately 40 proteins participated in electron and ion transfer, redox processes and stress response were also upregulated in the double mutant in comparison to the wild type and rescued to wild type-status when the AHL mixture was added (Figure 6B; Supplementary Table S5). Additionally, a category of proteins associated with invasiveness and toxicity was represented by metallopeptidases, aminopeptidases and proteases, and three enzymes associated with antibiotic resistance, i.e., DNA gyrase, beta-lactamase OXA-488 and protease PfpI. Lastly, an assembly of 18 proteins crucial for motility was represented by those involved in flagella-driven chemotaxis and piliation (Figure 6B; Supplementary Table S6).

At the same time, only nine proteins responsible for different functions were not significantly changed in the extracellular space of the double mutant in comparison to wild-type bacteria according to the ANOVA test (Figure 6A; Supplementary Tables S1, S3, S4, S6). These were elastase LasB, T3SS effector bifunctional cytotoxin exoenzyme T, chitin-binding protein CbpD and flagellar basal-body rod protein FlgF (Supplementary Table S6). Interestingly, Student’s *t*-test revealed a significant decrease in LasB protein abundance in the double mutant in comparison to wild-type bacteria, both treated with DMSO diluent (*p* = 0.031, fold change = 0.1). After treatment with AHL mixture, the decrease in LasB abundance in the double mutant in comparison to wild-type bacteria was not statistically significant (*p* = 0.12, fold change = 0.2) according to Student’s *t*-test.

In summary, our results here demonstrate that an extensive ensemble of extracellular proteins in *P. aeruginosa* PA14 is controlled by the LasI/RhlI system.

## Discussion

Quorum sensing (QS) system for intercellular communication is employed by numerous clinically significant bacteria to regulate processes related to pathogenicity and development of biofilms. QS signaling enables bacterial populations to coordinate collective responses, such as actions against competing microorganisms or host immune defenses, when cell density reaches a critical threshold. Employing this strategy, bacteria control the production of a large array of virulence factors, facilitate evasion of hosts, and enhance survival and tolerance to drugs.

**Figure 7:**
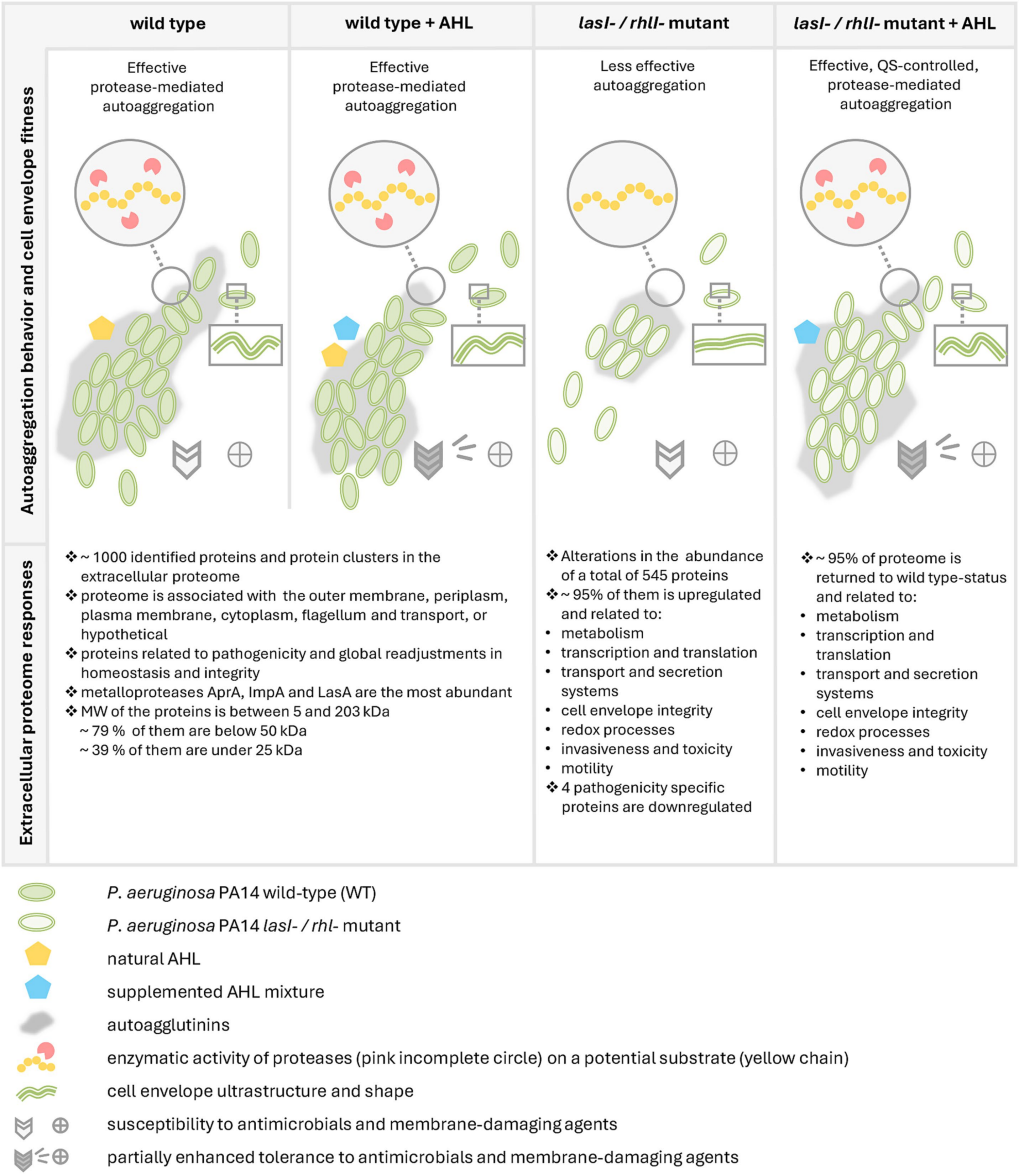
Overview of the study on the impact of *P. aeruginosa* PA14 LasI/RhlI quorum sensing system on protease-mediated autoaggregation behavior, cell envelope characteristics, and extracellular proteome responses. In the upper panel, the scheme illustrates the four main conditions for *P. aeruginosa* PA14 as an experimental model used in this study: wild-type bacteria; wild-type bacteria exposed to AHL mixture (wild-type + AHL); double mutant *lasI-/rhlI-*; and double mutant supplemented with AHL mixture (*lasI-/rhlI-* + AHL). In the middle panels, the scheme summarizes bacterial behavior to form suspended autoaggregates where the functional AHL-based LasI/RhlI system of QS network and protease activity act synergistically. Further, cell envelope characteristics are illustrated. The ultrastructure of the cell envelope is also dependent on an intact LasI/RhlI system. AHL-based QS signaling may provide bacteria with a partial tolerance to membrane-damaging and antimicrobial agents. The lower panel reflects mechanistical insights and mapped alterations in the extracellular proteome responses. This includes substantially perturbed abundance of an array of components associated with a large number of different functional groups of proteins playing a crucial role in pathogenicity-specific and global homeostatic processes.

In this study, using *P. aeruginosa* PA14 as a cell model, we observed a crucial contribution of the LasI/RhlI QS system to the protease-mediated program of multicellular community autoaggregation that occurs in fluids (Figure 7). This QS system appeared to control cell envelope characteristics, including morphology and tolerance to membrane-damaging and antimicrobial agents. Moreover, employing a quantitative proteomic approach, we demonstrate that the LasI/RhlI QS system perturbs the extracellular proteome in the late exponential and early stationary growth phase. Remarkably, about 95% of this extracellular proteome was upregulated in *lasI^−^/rhlI^−^* mutant in comparison to the wild type and rescued to wild-type status when the AHL mixture was added (Figures 6, 7). The proteins were associated with the outer membrane, periplasm, plasma membrane, cytoplasm, flagellum, and secretion machinery (Figure 6). The ensemble of events may reflect that bacteria are actively releasing a large amount of diverse proteins into the environment, e.g., via TSS and secretion of outer membrane vesicles. It is furthermore likely a consequence of bacterial adaptation to environmental conditions which includes acquiring nutrients, forming communities for protection and communication, and managing stress. *P. aeruginosa* PA14 is assumed to represent a clinically relevant, highly virulent strain, albeit displaying modest surface attachment for biofilm formation and a pronounced planktonic surface characteristics to facilitate the attachment of neighbors (Grace et al., 2022). Our results corroborate previous findings on transcriptomic and metabolomic effects (Schuster and Greenberg, 2006; Schuster et al., 2003; Davenport et al., 2015), and proteomic alterations, where *P. aeruginosa* PAO1 was used as a model (Arevalo-Ferro et al., 2003; Nouwens et al., 2003), and include revealing common biological processes, such as pathogenicity, global metabolism, transcription, stress tolerance, and key elements of regulation.

We found that wild-type *P. aeruginosa* PA14 possesses a more effective ability to form suspended autoaggregates than its *lasI^−^/rhlI^−^* mutant defective in the production of QS signal molecules 3O-C_12_-HSL and C_4_-HSL (Figure 1). When AHL mixture was added to *lasI^−^/rhlI^−^* mutant, the level of autoaggregation was returned to wild-type status, which points to the QS control of the process (Figure 1). Along with the AHL-based QS circuits, protease activity has an impact on aggregation in *P. aeruginosa*. However, it did not appear to require eDNA, suggesting that this scaffold may play a minor role in this event in our experimental model (Figures 2, 3). Bacterial aggregation is assumed to occur primarily in the period between late exponential and early stationary phases when most QS-regulated proteins are induced (Arevalo-Ferro et al., 2003; Sauvage and Hardouin, 2020; Scott et al., 2013). The effect of protease activity on aggregation is in line with our data on extracellular proteome, where we identified four proteins associated with enzymatic proteolysis of host connective tissues and thereby invasiveness, namely the metalloproteases AprA, ImpA and LasA, and alkaline protease secretion protein AprF, all being downregulated in the double mutant in comparison to wild type and rescued to wild type-status when AHL was added (Figure 6; Supplementary Table S6). Two proteins, AprA and LasA, and the *lasA* gene, have been reported to be QS-controlled in proteomic and transcriptomic studies (Nouwens et al., 2003; Schuster et al., 2003). It should be mentioned that proteases may trigger autoaggregation through several potential strategies: by cleaving a substrate crucial for the process; by degrading a repressor that inhibits aggregation; or by catalyzing the self-assembly of the biopolymeric nets that assist the process (Figure 7).

In natural environments and during infection, suspended bacterial aggregates are so prevalent that they have been proposed to represent a third distinct mode of bacterial growth, alongside the well-characterized biofilm and planktonic lifestyles. The autoaggregation process driven by the attachment between neighboring bacteria living as plankton is independent of the program for surface-associated biofilm establishment (Cai, 2020). However, free-floating aggregates may still have a profound impact on biofilm development. When landing on a surface, they might outcompete the biofilm population arising from single cells and subsequently dominate in further biofilm development (Kragh et al., 2016).

In general, our results strengthen previous observations that cell-surface-associated and extracellular components are important for bacterial autoaggregation during different growth phases. The unique environment in the cystic fibrosis lung appears enriched in hyperpiliated and autoaggregative *P. aeruginosa* small-colony variants (Haussler et al., 2003). In *P. aeruginosa* PAO1, cell-associated polysaccharide PsI is responsible for exponential phase aggregation, while both PsI and eDNA mediate this process in stationary phase cultures (Melaugh et al., 2023); eDNA is also associated with late-stage biofilm establishment (Whitchurch et al., 2002; Ma et al., 2006). In the present study, we have delineated the complementary, protease-dependent mechanism of autoaggregation that does not require eDNA scaffolding. Although here we focused on the PA14 strain, and several preconditions represented by choice of media, growth phase, setting time and concentration, the finding might shed light on mechanisms of the process in other *P. aeruginosa* strains and conditions. Highly virulent PA14 displays less initial progress to surface attachment, driven by the cell-associated polysaccharide Pel that facilitates the attachment of neighbors. PA14 forms relatively weak biofilms through the Chp pathway, which adjusts the cAMP concentration to promote planktonic surface memory. By contrast, the comparatively less virulent PAO1 demonstrates rapid attachment to surfaces and progression to biofilm formation through the Wsp pathway for surface recognition and larger production of exopolysaccharides, promoting attachment (Lee et al., 2020). In another gram-negative pathogen, *Vibrio cholerae*, several proteases and eDNA are important for a rapid and QS-controlled program of suspended aggregation independently of the surface-associated biofilm development (Detomasi et al., 2023; Jemielita et al., 2018; Jemielita et al., 2021).

In *P. aeruginosa*, the QS system regulates the expression of a diverse array of molecules, including cell surface-associated and secreted proteins, which potentially may influence cell surface architecture and overall bacterial fitness. Furthermore, the envelope is an important drug target and determinant of intrinsic drug resistance. We, therefore, focused further investigations on cell envelope morphology and tolerance to membrane-damaging and antimicrobial agents. Employing high-resolution imaging and phenotypic assays, we demonstrated that the ultrastructural characteristics of the cell envelope in *P. aeruginosa* PA14, including its shape and curvature pattern, as well as tolerance to membrane-damaging and antimicrobial agents, are controlled by the AHL-dependent LasI/RhlI system (Figures 4, 5). Thus, in wild-type bacteria treated with either diluent or AHL, the diderm cell envelope appeared well structured with pronounced and regular undulations. In contrast, in the *lasI^−^/rhlI^−^* mutant treated with diluent, it appeared less folded. However, the double mutant regained the bending pattern of the envelope when AHL was added, speaking for the QS control of the process (Figure 4). Cell envelope curvature may serve as a marker not only for cell envelope topography, but also for fitness and drug tolerance. Indeed, in Gram-negative bacteria, alterations in cell envelope curvature depend on the lipid composition and remodeling, protein insertions and scaffolding, enzymatic modifications and accumulation of compounds in the periplasmic space (Lithgow et al., 2023). In contrast to eukaryotes, relatively few membrane remodeling systems and curvature-mediating proteins have been characterized in detail. Remodeling of the envelope is essential for proper division, to facilitate infection and response to environmental changes (Bohuszewicz et al., 2016). Moreover, we found that wild-type and *lasI^−^/rhlI^−^* mutant showed similar levels of susceptibility to membrane-damaging and antimicrobial agents (Figure 5). The addition of AHL provided partial tolerance to the membrane-damaging agents and enhanced resistance to some antimicrobial drugs that use porin- or lipid-mediated uptake to enter the cell. There is experimental evidence to suggest that the complexity, dynamics, and remodeling of bacterial membrane architecture can influence drug tolerance within populations of *P. aeruginosa, Burkholderia cenocepacia, Escherichia coli, Staphylococcus aureus,* and *Mycobacterium tuberculosis* (Lapinska et al., 2022; Lithgow et al., 2023; Larrouy-Maumus et al., 2016).

The morphology and biogenesis of the complex multilayered cell envelope of Gram-negative bacteria is coordinated by interconnected biosynthetic pathways and assembly machines to ensure their integrity and protection as well as trans-envelope transport and secretion (Silhavy et al., 2010; Fivenson et al., 2024). Using a quantitative proteomic approach, we mapped large ensembles of proteins allocated to cell envelope integrity that were upregulated in the double mutant in comparison with the wild type and rescued to wild-type status when AHL was added (Figure 6; Supplementary Table S4). Among them, we categorized the groups with the following functions: envelope synthesis, support and remodeling; outer membrane structure and assembly; envelope regulation and adaptation; and Tol-Pal system for envelope maintenance. The Tol-Pal system is a set of protein complexes that traverse the outer membrane, periplasmic space, and inner membrane. Mutations in *tol/pal* genes have revealed that they are critical for growth, outer membrane integrity and susceptibility to some surface-active chemicals and antimicrobial agents, e.g., to colistin and *β*-lactams (Lo Sciuto et al., 2014; Hirakawa et al., 2022; Llamas et al., 2000). In the outer membrane, porins facilitate nutrient uptake and drug entry into the bacterial cell. In *P. aeruginosa*, the relative dearth of porins within the outer membrane and a large set of specific porins OprD, that permit the entry of only a few molecules, decrease the rate at which antibiotics can penetrate the cell. The Opr and OmpA families have mainly structural roles. Still, unexpectedly efficient porin-independent traffic may occur through the outer membrane for diverse compounds (Poole, 2011; Ude et al., 2021). Yet another set included proteins involved in cell envelope synthesis, support and remodeling (Supplementary Table S4). Synthesis pathways for major envelope components, phospholipids, peptidoglycan, and lipopolysaccharide (LPS), have common precursors. Sugar UDP-N-acetylglucosamine is used to form peptidoglycan in the cell wall as well as the carbohydrates in the lipid A component of LPS. Acyl carrier protein provides acyl chains for LPS and phospholipids. Long- and short-chain acyl groups enter phospholipids and LPS synthesis, respectively. Lpx family enzymes are key nodes of envelope synthesis regulation, and Lpt pathway coordinates LPS transport to the outer membrane (Fivenson et al., 2024). Here, we found that the AHL-dependent LasI/RhlI system has a profound impact on cell envelope integrity in *P. aeruginosa* PA14. These findings corroborate our data on the ultrastructure of the cell envelope and tolerance to membrane-damaging and antimicrobial agents (Figures 4, 5).

Beyond the link between QS and cell envelope integrity, our data show that a large group of proteins associated with transport and secretion systems were upregulated in *lasI^−^/rhlI^−^* mutant in comparison to the wild type and returned to wild-type status when AHL was added (Figure 6; Supplementary Table S3). These changes may occur in the extracellular space due to gene expression changes, transport and secretion activities, and the trafficking of outer membrane vesicles (Sauvage and Hardouin, 2020; Dou et al., 2024). Within this group, we identified the following: ABC and TRAP transporters; secretion system proteins; TonB transporters; porins, efflux and outer membrane proteins; iron uptake and metal ion binding. Several transport families, like the ATP-binding cassette (ABC) and tripartite ATP-dependent periplasmic (TRAP), employ solute-binding proteins (SPB) to capture the substrate in the extracytosolic space and present it to transmembrane permeases. These transporters facilitate critical processes through the translocation of multiple substrates across cellular membranes and, thereby, play a vital role in pathogenesis via the import of essential molecules as well as the export of toxic and destructive virulence traits. Moreover, ABC exporters support bacterial pathogenicity by exporting toxic substances, thus contributing to the development of drug resistance (Akhtar and Turner, 2022; Matilla et al., 2021). *P. aeruginosa* harbors a resistance-nodulation-division (RND) family network that includes efflux systems (MexAB-OprM, MexCD-OprJ, MexEF-OprN and MexXY), and their overexpression not only contributes to antibiotic resistance but may also affect physiology, for example, by shutting down the QS-response and production of QS-controlled virulence factors (Alcalde-Rico et al., 2018). The Mla transport system maintains lipid asymmetry and influences outer membrane dynamics (Munguia et al., 2017).

Several enzymes associated with antibiotic resistance, i.e., DNA gyrase, beta-lactamase OXA-488 and protease PfpI, were upregulated in the double mutant in comparison with wild type but rescued to wild-type status when AHL was added (Supplementary Table S6). DNA gyrase is the target of fluoroquinolones, and resistance to this drug may occur via target site mutations or altered expression, often in combination with efflux changes. Resistance to β-lactams may arise from upregulation of the intrinsic cephalosporinase AmpC, acquisition of transferable β-lactamases, increased drug efflux via MexAB-Opr systems, or outer membrane impermeability via changes in porin OprD (Poole, 2011). Bacterial proteases, e.g., PfpI and others, are known to play essential roles in orchestrating antibiotic resistance, swarming motility, and biofilm formation (Fernandez et al., 2012).

The increased levels of two remarkably large groups of proteins for metabolism (Figure 6; Supplementary Table S1) and for transcription and translation (Figure 6; Supplementary Table S2) were also identified. Here, we categorized sets comprising the essential components of RNA and DNA polymerases, regulatory factors, and structural 30S and 50S ribosomal proteins. Since up to 10% of the large *P. aeruginosa* genome is controlled by QS (Schuster and Greenberg, 2006), it is obvious that QS impacts other key elements of regulation, e.g., QSregVF, CsrA, BolA, MvaU, and CigR. Moreover, it is not surprising that even the ribosome translational machinery is controlled by QS (Supplementary Table S2). Indeed, several levels of QS-control of bacterial metabolism and transcriptional regulation have been reported and reviewed (Rutherford and Bassler, 2012; Goo et al., 2015). Manipulated expression of proteins for metabolism, transcription, translation and accessory regulators may fine-tune the QS cross-talk and cooperatively support metabolic homeostasis and dynamics. These occurrences may suggest that the production of virulence traits occurs with distinct precision. The events may also stand as a contributing factor that affects resistance to antimicrobial agents that block DNA production and protein synthesis via ribosome. These findings may also explain our data on susceptibility to antimicrobial agents (Figure 5).

A pronounced link between QS and stress tolerance was demonstrated by mapping a large set of proteins related to electron and ion transfer, redox processes and stress response (Figure 6; Supplementary Table S5). Indeed, *P. aeruginosa*, as electrogenic bacteria, operate extracellular electron transfer pathways to maintain their redox state, respiration, and exchange energy with their surroundings. Bacterial cytochromes, nanowires and phenazines, like pyocyanin, facilitate the transfer of electrons to and from other molecules. In multicellular populations, eDNA binds to pyocyanin, enhancing electron transfer within the bacterial community (Saunders et al., 2020). A group of QS-regulated genes associated with stress tolerance was suggested in transcriptome analyses (Schuster et al., 2003; Wagner et al., 2003). Under oxidative stress, planktonic bacteria overexpress genes that are crucial for QS communication, redox processes, survival, virulence and biofilms (Kim et al., 2024). QS-related boost of stress tolerance should allow bacteria to counteract QS hampering and invasions by environmental competitors, having an impact on bacterial ecology (Garcia-Contreras et al., 2015).

The Las and Rhl systems are tightly linked to each other, where signaling of 3O-C_12_-HSL and C_4_-HSL is likely needed for shared maximal effect (Deziel et al., 2004; Schuster et al., 2003; Miranda et al., 2022; Thomas et al., 2025). Together, they control a specific outcome, such as the expression of a virulence trait or shared phenotype, which is triggered to an optimal level when both AHL-dependent signals are present (Thomas et al., 2025; Schuster et al., 2003). While the focus of this study was to assess the pathogenic potential of *P. aeruginosa* by investigating the shared contribution of LasI/RhlI AHL-dependent QS system, some experimental sets included *lasI-* and *rhlI-* single mutants. Still, the individual roles of LasI and RhlI were not analyzed but remain important for understanding the mechanisms underlying QS-mediated bacterial behavior and will require further attention in a follow-up study. Indeed, whereas it is informative to explore the contribution of the entire Las/Rhl QS system (Rumbaugh et al., 1999; Holm et al., 2015), investigations of Las and Rhl circuits in isolation and specific environmental context (Simanek et al., 2023; Mukherjee et al., 2017), even though they are challenging, also provide valuable information on their individual roles and impact on *P. aeruginosa* behaviors.

In conclusion, the results provide insight into how bacteria utilize QS-controlled protease activity to form aggregates as a lifestyle form and adjust their proteome for optimal survival and intercellular communication (Figure 7). Our study demonstrates that the LasI/RhlI system of QS network controls *P. aeruginosa* PA14 behavior to form suspended autoaggregates in a protease-mediated way and thereby the cooperation within a multifaceted microbial community. This AHL-dependent QS circuit also impacts the ultrastuctural and fitness characteristics of the cell envelope, namely its curvature pattern, and tolerance to membrane-damaging and antimicrobial agents. Moreover, this was substantiated in a sophisticated and multifactorial way by a perturbed abundance of an array of components in the secreted proteome associated with many different functional groups of proteins playing a pivotal role in both pathogenicity-specific processes and global homeostatic adjustments within cooperative bacterial populations (Figure 7). This control system appears to be crucial as a bacterial survival and adaptation strategy, since if it runs out of governing, or is weakened, it would be negative to the long-term sustainability of the microbial communities but is turned on when necessary. These findings might have potential clinical implications, as manipulations of the QS system serve as a therapeutic strategy to disrupt microbial communities and reduce bacterial virulence. Understanding how QS is regulated at the proteomic level offers new insights into bacterial responses to environmental changes and pathogenicity. This opens avenues for targeted interventions aimed at modulating QS signaling to combat antibiotic resistance and infections caused by opportunistic pathogens such as *P. aeruginosa*.

## Data Availability

The mass spectrometry proteomics data are available in the ProteomeXchange Consortium via the PRIDE partner repository with the dataset identifier PXD071007.

## Footnotes

1
https://www.ncbi.nlm.nih.gov/

2
https://www.uniprot.org/

## Glossary

3O-C_12_-HSL: *N*-3-oxo-dodecanoyl-L-homoserine lactone
C_4_-HSL: *N*-butyryl-L-homoserine lactone
AHL: *N*-acylhomoserine lactone
A280, A450 and A600: absorbances at 280, 450 and 600 nm wavelength, respectively
CFU/ml: colony-forming units per milliliter
CS: cell culture supernatant
DMSO: dimethylsulfoxide
dsDNA: double stranded DNA
eDNA: extracellular DNA
DTT: dithiothreitol
EDTA: ethylenediaminetetraacetic acid
IAA: iodoacetamide
LB: Luria-Bertani medium
LC–MS/MS: liquid chromatography tandem mass spectrometry
LPS: lipopolysaccharide
MH: Mueller Hinton medium
MIC: minimal inhibitory concentration
MINS: minimal medium
MOPS: 3-morpholinopropane sulfonic acid
MW: molecular weight
OM: outer membrane
P: periplasmic space
PM: plasma membrane
RT: room temperature
SDS: sodium dodecyl sulfate
SS: secretion systems
T1SS to T6SS: type 1, 2, 3, 4, 5 and 6 secretion systems
TNBSA: trinitrobenzene sulfonic acid
TEM: transmission electron microscopy
QS: quorum sensing
