# Supplementary material for: Pseudomonas aeruginosa LasI/RhlI quorum sensing system controls protease-mediated autoaggregation behavior, cell envelope characteristics and extracellular proteome responses
Source: Front Microbiol. 2026 Jan 5;16:1693814. doi: 10.3389/fmicb.2025.1693814 (PMC12812693; doi:10.3389/fmicb.2025.1693814)
Supplement: Supplementary file 1 [file Data_Sheet_1.docx]

***Supplementary Material***

***Pseudomonas aeruginosa* LasI/RhlI quorum sensing system controls protease-mediated autoaggregation behavior, cell envelope characteristics and** **extracellular proteome responses**

**Albin Eriksson ^1^, Maria V. Turkina ^1^, Maria Ntzouni ^2^, Karl-Eric Magnusson ^1^,**

**Elena Vikström ^1^***

***^1^*** *Department of Biomedical and Clinical Sciences, Faculty of Medicine and Health Sciences, Linköping University, Sweden,*

***^2^*** *Core Facility, Faculty of Medicine and Health Sciences, Linköping University, Sweden*

* Correspondence:

Elena Vikström

Linköping University

Faculty of Medicine and Health Sciences

Department of Biomedical and Clinical Sciences

Linköping SE-581 85 Sweden

[elena.vikstrom@liu.se](mailto:elena.vikstrom@liu.se)

Total number of supplementary figures, tables and text: 7

(Including 6 tables and 1 figure)

**FIGURES**

**
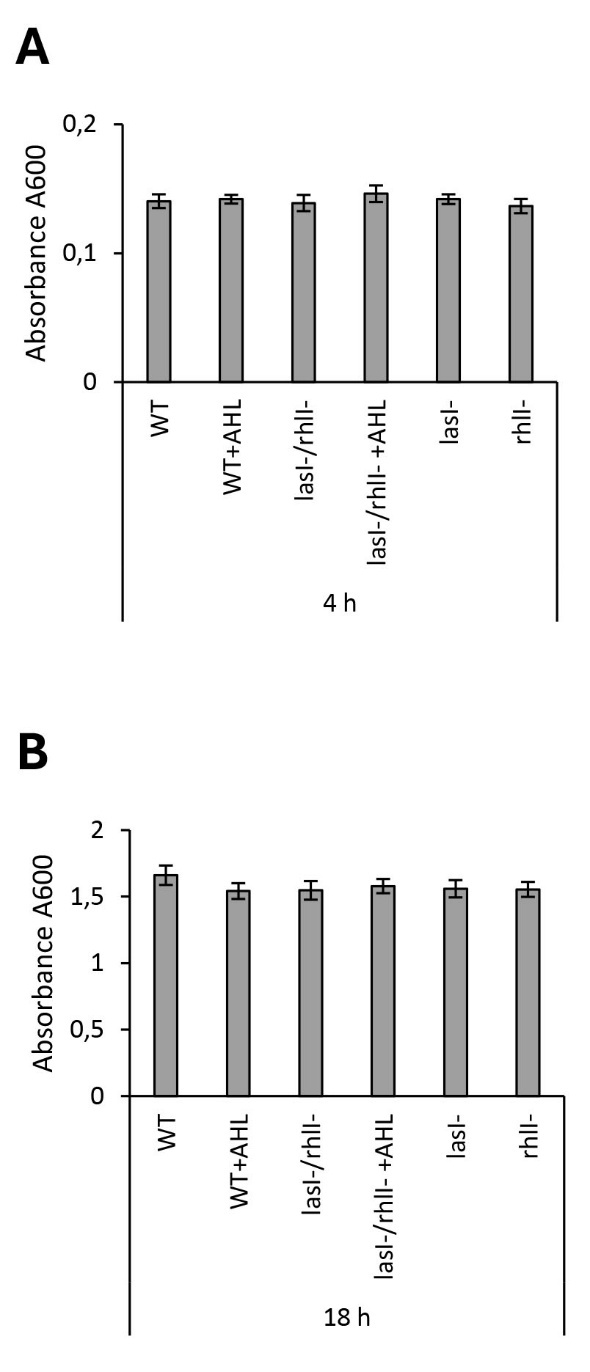
**

**Figure S1.** The growth of four *P. aeruginosa* PA14 strains that were used in this study. Wild-type, double mutant *lasI-/rhlI-* and single mutants *lasI-* and *rhlI-* were resuspended in a defined minimal medium MINS to A600 of 0.1 and further diluted with MINS to initial absorbance of 0.015 at 600 nm (A600). Wild-type bacteria (WT), *lasI-/rhl-*, *lasI-* and *rhlI-* mutants were treated with DMSO as a diluent control. A mixture of 5 μM 3O-C_12_-HSL and 5 μM C_4_-HSL was added to wild-type (WT+AHL) and double mutant (*lasI-/rhlI-* +AHL). Bacteria were grown at 37° with shaking and aeration. Bacterial growth was quantified in (**A**) 4-h and (**B**) 18-h cultures by measuring A600. At least 4 independent experiments in triplicates were performed on separate days on different bacteria cultures. Columns represent the means ± SE. The growth patterns of the strains were similar within 4- and 18-h time points.

**TABLES**

**Table S1.** Selected groups of proteins differentially expressed in wild-type *P. aeruginosa* PA14 and its *lasI-/rhlI-* mutant exposed to AHL mixture or treated with DMSO as a diluent control; the proteins are overall allocated to the functional group **metabolism** according to NCBI GO analyses.

| **Identified proteins** | **NCBI accession number** | **MW kDa** | ***p*-value** | **Quantitative profile *** |
| --- | --- | --- | --- | --- |
| **Metabolism** | | | | |
| **Central carbon metabolism and energy** | | | | |
| 2,3-bisphosphoglycerate-independent phosphoglycerate mutase | UXO63272.1 | 56 | 0.0014 | **X** |
| 3-phosphoglycerate kinase | UXO53661.1 | 11 | < 0.00010 | **X** |
| aconitate hydratase AcnA | UXO55509.1 | 99 | 0.00024 | **X** |
| citrate synthase | UXO31886.1 | 48 | < 0.00010 | **X** |
| class II fumarate hydratase | UXO32617.1 | 49 | 0.010 | **X** |
| fumarate hydratase | WP_003093938.1 | 55 | 0.00091 | **X** |
| fumarate hydratase class II 1 | EOT22018.1 | 49 | < 0.00010 | **X** |
| malate synthase G | EOT10524.1 | 79 | < 0.00010 | **X** |
| malic enzyme | EOT06748.1 | 45 | < 0.00010 | **X** |
| phosphoenolpyruvate carboxykinase | UXO51533.1 | 56 | < 0.00010 | **X** |
| phosphoenolpyruvate synthase | UXO55297.1 | 86 | < 0.00010 | **X** |
| pyruvate dehydrogenase (acetyl-transferring), homodimeric type | UXO57254.1 | 100 | < 0.00010 | **X** |
| pyruvate kinase | UXO32858.1 | 52 | < 0.00010 | **X** |
| succinate dehydrogenase flavoprotein subunit | EOT21323.1 | 64 | < 0.00010 | **X** |
| succinate--CoA ligase subunit alpha | WP_003087428.1 | 30 | < 0.00010 | **X** |
| NADP-dependent isocitrate dehydrogenase | UXO36566.1 | 46 | 0.00083 | **X** |
| NADP-dependent succinate-semialdehyde dehydrogenase | UXO34472.1 | 52 | 0.00064 | **X** |
| **Fatty acid and lipid metabolism** | | | | |
| acyl carrier protein | UXO42110.1 | 9 | 0.024 | **X** |
| ACP S-malonyltransferase | UXO36205.1 | 32 | < 0.00010 | **X** |
| beta-ketoacyl-ACP synthase I | UXO49559.1 | 43 | < 0.00010 | **X** |
| beta-ketoacyl-ACP synthase II | UXO48011.1 | 43 | < 0.00010 | **X** |
| 3-oxoacyl-ACP reductase FabG | UXO59811.1 | 26 | 0.018 | **X** |
| enoyl-ACP reductase FabV | UXO59828.1 | 44 | 0.00012 | **X** |
| acetyl-CoA C-acyltransferase FadA | WP_003138585.1 | 42 | < 0.00010 | **X** |
| fatty acid oxidation complex subunit alpha FadB | WP_003091204.1 | 77 | < 0.00010 | **X** |
| acetyl-CoA carboxylase biotin carboxylase subunit | UXO62990.1 | 49 | 0.018 | **X** |
| **Nucleotide and cofactor metabolism** | | | | |
| 5-(carboxyamino)imidazole ribonucleotide mutase | UXO51760.1 | 17 | 0.00014 | **X** |
| 5-(carboxyamino)imidazole ribonucleotide synthase | UXO51759.1 | 39 | 0.00075 | **X** |
| 5-methyltetrahydropteroyltriglutamate-homocysteine methyltransferase | EOT16886.1 | 86 | 0.0012 | **X** |
| AMP nucleosidase | UXO52871.1 | 56 | < 0.00010 | **X** |
| adenosylhomocysteinase | UXO64146.1 | 51 | < 0.00010 | **X** |
| adenosylmethionine decarboxylase | UXO58461.1 | 31 | 0.0021 | **X** |
| adenylate kinase | UXO53190.1 | 23 | 0.0088 | **X** |
| adenylosuccinate lyase | UXO42462.1 | 51 | 0.0012 | **X** |
| adenylosuccinate synthase | UXO63078.1 | 47 | < 0.00010 | **X** |
| carbamoyl-phosphate synthase large chain | EOT07046.1 | 117 | 0.00036 | **X** |
| CTP synthase | UXO35536.1 | 60 | < 0.00010 | **X** |
| orotate phosphoribosyltransferase | UXO63472.1 | 23 | 0.0045 | **X** |
| phosphoribosylamine--glycine ligase | UXO57096.1 | 45 | 0.0011 | **X** |
| phosphoribosylformylglycinamidine cyclo-ligase | UXO44326.1 | 37 | 0.0023 | **X** |
| pyridoxamine 5'-phosphate oxidase | UXO61935.1 | 25 | 0.0030 | **X** |
| **Carbohydrate metabolism** | | | | |
| fructose-bisphosphate aldolase class II | WP_003084964.1 | 39 | 0.00054 | **X** |
| gluconolactonase PpgL | UXO34943.1 | 42 | 0.0026 | **X** |
| ribose-5-phosphate isomerase A | EOT10679.1 | 24 | 0.0010 | **X** |
| ribulose-phosphate 3-epimerase | UXO64319.1 | 24 | 0.00012 | **X** |
| transketolase | UXO40653.1 | 72 | 0.00043 | **X** |
| type I glyceraldehyde-3-phosphate dehydrogenase | UXO41895.1 | 36 | 0.0057 | **X** |
| phosphoheptose isomerase | UXO38854.1 | 21 | 0.0011 | **X** |
| phosphopyruvate hydratase | UXO65044.1 | 45 | < 0.00010 | **X** |
| **Proteases and peptidases** | | | | |
| ATP-dependent protease | EOT21806.1 | 90 | 0.00065 | **X** |
| ATP-dependent zinc protease | UXO58272.1 | 19 | 0.00029 | **X** |
| periplasmic tail-specific protease | EOT15007.1 | 79 | 0.0031 | **X** |
| protease IV | UXO58572.1 | 48 | < 0.00010 | **X** |
| protease PfpI | UXO34563.1 | 19 | 0.013 | **X** |
| TIGR02281 family clan AA aspartic protease | UXO52796.1 | 23 | 0.00056 | **X** |
| Zn-dependent protease with chaperone function | EOT10734.1 | 27 | 0.013 | **X** |
| ATP-dependent endopeptidase proteolytic subunit ClpP | UXO37566.1 | 24 | < 0.00010 | **X** |
| proteolytic complex protein CptA | UXO63275.1 | 46 | 0.012 | **X** |
| aminopeptidase PaaP | UXO65740.1 | 58 | 0.0051 | **X** |
| M14 family metallopeptidase | UXO42023.1 | 71 | 0.0012 | **X** |
| methionine aminopeptidase, type I | EOT11349.1 | 29 | 0.0021 | **X** |
| neutral zinc metallopeptidase | UXO51058.1 | 32 | 0.018 | **X** |
| osmoprotectant NAGGN system M42 family peptidase | UXO59299.1 | 43 | 0.012 | **X** |
| signal peptide peptidase SppA | UXO65706.1 | 36 | 0.00090 | **X** |
| **Chaperones, protein folding and assembly** | | | | |
| chaperonin GroEL | UXO38814.1 | 57 | 0.012 | **X** |
| co-chaperone GroES | UXO50617.1 | 10 | 0.00066 | **X** |
| chaperone surA | EOT10411.1 | 48 | < 0.00010 | **X** |
| Hsp33 family molecular chaperone HslO | UXO45633.1 | 33 | 0.0058 | **X** |
| molecular chaperone DnaK | UXO57004.1 | 68 | < 0.00010 | **X** |
| ClpB | AAP81264.1 | 95 | 0.012 | **X** |
| peptidylprolyl isomerase | WP_003087890.1 | 18 | 0.00026 | **X** |
| peptidylprolyl isomerase PpiA | WP_003109185.1 | 20 | < 0.00010 | **X** |
| ribosome maturation factor RimP | WP_003095193.1 | 17 | 0.016 | **X** |
| ribosome recycling factor | UXO53223.1 | 20 | < 0.00010 | **X** |
| nucleotide exchange factor GrpE | UXO51103.1 | 21 | < 0.00010 | **X** |
| bifunctional protein-disulfide isomerase/oxidoreductase DsbC | UXO35435.1 | 26 | 0.026 | **X** |
| **Amino acid metabolism and synthesis** | | | | |
| 2,3,4,5-tetrahydropyridine-2,6-dicarboxylate N-succinyltransferase | UXO41410.1 | 36 | 0.013 | **X** |
| 2-isopropylmalate synthase | UXO41248.1 | 62 | 0.00022 | **X** |
| 3-isopropylmalate dehydrogenase | UXO47862.1 | 39 | < 0.00010 | **X** |
| 3-methyl-2-oxobutanoate hydroxymethyltransferase | UXO33368.1 | 28 | 0.0055 | **X** |
| 4-aminobutyrate--2-oxoglutarate transaminase | UXO34473.1 | 45 | 0.00014 | **X** |
| 4-hydroxy-tetrahydrodipicolinate synthase | UXO61976.1 | 31 | < 0.00010 | **X** |
| 2-methylcitrate synthase | UXO44480.1 | 42 | 0.00039 | **X** |
| 2-oxoglutarate dehydrogenase complex dihydrolipoyllysine-residue succinyltransferase | WP_011666712.1 | 43 | 0.00086 | **X** |
| 3-hydroxyacyl- | UXO43657.1 | 19 | < 0.00010 | **X** |
| 3-phosphoshikimate 1-carboxyvinyltransferase | EOT14892.1 | 79 | 0.00019 | **X** |
| acetate kinase | UXO50336.1 | 42 | 0.013 | **X** |
| adenosylmethionine decarboxylase | UXO58461.1 | 31 | 0.0021 | **X** |
| adenylate kinase | UXO53190.1 | 23 | 0.0088 | **X** |
| ADP-forming succinate--CoA ligase subunit beta | UXO37779.1 | 42 | 0.00030 | **X** |
| agmatine deiminase | UXO40400.1 | 41 | 0.011 | **X** |
| agmatine deiminase family protein | UXO44034.1 | 41 | 0.00033 | **X** |
| alginate O-acetyltransferase AlgF | UXO35621.1 | 23 | < 0.00010 | **X** |
| alkanesulfonate monooxygenase | EOT11152.1 | 42 | 0.014 | **X** |
| alpha/beta fold hydrolase | WP_003109177.1 | 51 | 0.0090 | **X** |
| alpha/beta hydrolase | UXO43582.1 | 35 | 0.0071 | **X** |
| alpha/beta hydrolase family protein | UXO40707.1 | 38 | 0.0098 | **X** |
| amidase | UXO40880.1 | 60 | 0.012 | **X** |
| aminodeoxychorismate/anthranilate synthase component II | UXO52554.1 | 22 | 0.026 | **X** |
| ammonia-dependent NAD(+) synthetase | UXO51258.1 | 30 | 0.0044 | **X** |
| anthranilate phosphoribosyltransferase | WP_003085203.1 | 37 | 0.00025 | **X** |
| anti-sigma factor MucA | UXO44514.1 | 21 | < 0.00010 | **X** |
| arginine deiminase | UXO45610.1 | 46 | 0.0024 | **X** |
| argininosuccinate lyase | UXO33901.1 | 52 | 0.0029 | **X** |
| arginyl-tRNA synthetase | EOT06743.1 | 65 | 0.00031 | **X** |
| asparaginase | UXO49824.1 | 39 | < 0.00010 | **X** |
| aspartate ammonia-lyase | UXO34060.1 | 51 | 0.020 | **X** |
| aspartate/tyrosine/aromatic aminotransferase | UXO36039.1 | 43 | < 0.00010 | **X** |
| aspartate/tyrosine/aromatic aminotransferase | UXO50303.1 | 43 | 0.0010 | **X** |
| aspartate-semialdehyde dehydrogenase | UXO59665.1 | 40 | 0.00042 | **X** |
| aspartyl/glutamyl-tRNA(Asn/Gln) amidotransferase subunit B | EOT22004.1 | 53 | 0.00036 | **X** |
| Asp-tRNA(Asn)/Glu-tRNA(Gln) amidotransferase subunit GatA | UXO33009.1 | 52 | < 0.00010 | **X** |
| bifunctional aconitate hydratase 2/2-methylisocitrate dehydratase | UXO49378.1 | 94 | < 0.00010 | **X** |
| bifunctional glutamate N-acetyltransferase/amino-acid acetyltransferase ArgJ | UXO38831.1 | 42 | < 0.00010 | **X** |
| bifunctional methylenetetrahydrofolate dehydrogenase/methenyltetrahydrofolate cyclohydrolase FolD | UXO61177.1 | 31 | < 0.00010 | **X** |
| bifunctional succinylornithine transaminase/acetylornithine aminotransferase | UXO62084.1 | 44 | < 0.00010 | **X** |
| BON domain-containing protein | UXO54893.1 | 12 | 0.0020 | **X** |
| branched-chain-amino-acid transaminase | UXO33647.1 | 34 | 0.013 | **X** |
| catalase KatA | UXO58519.1 | 56 | 0.0013 | **X** |
| Chain A, Activator of HSP90 ATPase | pdb\|8ES5\|A | 19 | < 0.00010 | **X** |
| chorismate mutase | UXO45624.1 | 20 | 0.0031 | **X** |
| chorismate synthase | UXO61288.1 | 39 | 0.0063 | **X** |
| cobaltochelatase subunit CobN | WP_003139813.1 | 140 | 0.0075 | **X** |
| deoxycytidine deaminase | UXO50875.1 | 20 | 0.014 | **X** |
| dienelactone hydrolase family protein | UXO32309.1 | 28 | 0.020 | **X** |
| ecotin | EOT14475.1 | 17 | < 0.00010 | **X** |
| enolase-phosphatase E1 | EOT18498.1 | 24 | 0.00030 | **X** |
| esterase family protein | UXO54487.1 | 58 | 0.0064 | **X** |
| fumarylacetoacetate hydrolase family protein | UXO34525.1 | 24 | 0.0017 | **X** |
| gamma-glutamyltransferase | UXO34569.1 | 62 | 0.0011 | **X** |
| gamma-glutamyltransferase | WP_003086881.1 | 60 | < 0.00010 | **X** |
| glutamate-1-semialdehyde 2,1-aminomutase | WP_003132522.1 | 45 | < 0.00010 | **X** |
| glutamate-5-semialdehyde dehydrogenase | UXO41036.1 | 45 | 0.0012 | **X** |
| glutamate--ammonia ligase | UXO33754.1 | 52 | 0.00084 | **X** |
| glutamine-hydrolyzing carbamoyl-phosphate synthase small subunit | UXO45198.1 | 41 | < 0.00010 | **X** |
| glutamine-hydrolyzing GMP synthase | UXO41272.1 | 58 | < 0.00010 | **X** |
| glycerophosphodiester phosphodiesterase | UXO48691.1 | 41 | 0.011 | **X** |
| glycine cleavage system aminomethyltransferase GcvT | WP_003136048.1 | 39 | 0.0063 | **X** |
| glycine cleavage system H protein 2 | EOT06575.1 | 15 | 0.0087 | **X** |
| HAD family phosphatase | UXO41812.1 | 24 | 0.015 | **X** |
| hemagglutinin repeat-containing protein | WP_016254199.1 | 532 | < 0.00010 | **X** |
| histidinol dehydrogenase | UXO62481.1 | 47 | < 0.00010 | **X** |
| HugZ family protein | UXO32916.1 | 27 | 0.022 | **X** |
| hydroxyisourate hydrolase | UXO55553.1 | 14 | 0.00051 | **X** |
| indole-3-glycerol phosphate synthase TrpC | UXO46655.1 | 30 | 0.0027 | **X** |
| inorganic diphosphatase | UXO35110.1 | 19 | 0.017 | **X** |
| isocitrate lyase | UXO54258.1 | 59 | 0.00095 | **X** |
| isoprenoid biosynthesis glyoxalase ElbB | WP_003099212.1 | 23 | 0.026 | **X** |
| leucyl-tRNA synthetase | EOT11724.1 | 98 | 0.0029 | **X** |
| low specificity L-threonine aldolase | UXO39945.1 | 38 | 0.0025 | **X** |
| MaoC family dehydratase | UXO46929.1 | 17 | 0.0018 | **X** |
| methionine adenosyltransferase | UXO34750.1 | 43 | 0.0051 | **X** |
| ornithine carbamoyltransferase | UXO35634.1 | 34 | < 0.00010 | **X** |
| orotate phosphoribosyltransferase | UXO63472.1 | 23 | 0.0045 | **X** |
| osmotically-inducible lipoprotein OsmE | WP_003095488.1 | 13 | 0.0056 | **X** |
| O-succinylhomoserine sulfhydrylase | UXO59675.1 | 43 | < 0.00010 | **X** |
| peptide chain release factor 2 | UXO47275.1 | 41 | 0.00065 | **X** |
| phenylalanyl-tRNA synthetase beta chain | EOT14450.1 | 87 | 0.020 | **X** |
| phosphate acetyltransferase | UXO56239.1 | 76 | 0.012 | **X** |
| phosphoglycerate kinase | UXO40657.1 | 40 | < 0.00010 | **X** |
| phosphoribosylformylglycinamidine synthase | WP_003137917.1 | 141 | < 0.00010 | **X** |
| porphobilinogen synthase | UXO33880.1 | 37 | 0.023 | **X** |
| PQQ-dependent sugar dehydrogenase | UXO55967.1 | 41 | 0.024 | **X** |
| putative hydro-lyase | UXO31362.1 | 29 | 0.0100 | **X** |
| putrescine--pyruvate aminotransferase | UXO52209.1 | 50 | 0.025 | **X** |
| pyochelin non-ribosomal peptide synthetase PchE | UXO40824.1 | 157 | 0.020 | **X** |
| queuine tRNA-ribosyltransferase | EOT11549.1 | 42 | < 0.00010 | **X** |
| salicyl-AMP ligase PchD | AAD55799.1 | 60 | 0.021 | **X** |
| serine hydroxymethyltransferase | UXO45038.1 | 45 | 0.0023 | **X** |
| serine--tRNA ligase | UXO42479.1 | 47 | 0.00040 | **X** |
| skp-like protein | EOT11339.1 | 19 | < 0.00010 | **X** |
| succinylglutamate desuccinylase | UXO62078.1 | 37 | 0.0012 | **X** |
| threonine ammonia-lyase, biosynthetic | EOT10678.1 | 55 | 0.00037 | **X** |
| threonine synthase | UXO35437.1 | 52 | < 0.00010 | **X** |
| transglutaminase family protein | UXO31422.1 | 70 | 0.00017 | **X** |
| tryptophan synthase alpha chain | EOT09839.1 | 28 | 0.015 | **X** |
| tryptophan synthase beta chain | EOT09838.1 | 44 | 0.0034 | **X** |
| uroporphyrinogen-III C-methyltransferase | UXO45698.1 | 41 | 0.0081 | **X** |
| valine--tRNA ligase | UXO47105.1 | 108 | 0.012 | **X** |
| YajQ family cyclic di-GMP-binding protein | UXO56528.1 | 18 | < 0.00010 | **X** |
| YegP family protein | UXO34536.1 | 12 | 0.00031 | **X** |
| YicC family protein | UXO57575.1 | 32 | < 0.00010 | **X** |
| zinc-binding alcohol dehydrogenase family protein | UXO35603.1 | 37 | 0.00020 | **X** |

*Quantitative profile

**X** - Downregulated in double mutant in comparison to wild type and rescued to wild type-status when AHL added.

**X** - Upregulated in double mutant in comparison to wild type and rescued to wild type-status when AHL added.

**X** - Upregulated in double mutant in comparison to wild type.

**X** - No changes in double mutant in comparison to wild type. Distinct effect of AHL in either wild type or double mutant or both.

**Table S2.** Selected groups of proteins differentially expressed in wild-type *P. aeruginosa* PA14 and its *lasI-/rhlI-* mutant exposed to AHL mixture or treated with DMSO as a diluent control; the proteins are overall allocated to the functional group **transcription and translation** according to NCBI GO analyses.

| **Identified proteins** | **NCBI**  **accession**  **number** | **MW**  **kDa** | **p-value** | **Quantitative profile *** |
| --- | --- | --- | --- | --- |
| **Transcription and translation** | | | | |
| **Components of RNA and DNA polymerases, regulatory factors** | | | | |
| RNA polymerase-binding protein DksA | UXO62867.1 | 17 | 0.019 | **X** |
| DNA-directed RNA polymerase subunit alpha | UXO46714.1 | 37 | < 0.00010 | **X** |
| DNA-directed RNA polymerase subunit beta | UXO52583.1 | 151 | 0.0050 | **X** |
| DNA-directed RNA polymerase subunit beta' | EOT10329.1 | 154 | 0.00054 | **X** |
| DNA-directed RNA polymerase subunit omega | UXO57577.1 | 10 | 0.0030 | **X** |
| sigma factor AlgU regulator MucB | UXO50414.1 | 35 | 0.0011 | **X** |
| anti-sigma factor MucA | UXO44514.1 | 21 | < 0.00010 | **X** |
| transcription elongation factor greA | EOT07047.1 | 17 | 0.00078 | **X** |
| transcription termination factor NusA | UXO51086.1 | 55 | 0.024 | **X** |
| translation initiation factor IF-1 | WP_002553999.1 | 8 | < 0.00010 | **X** |
| elongation factor P | UXO54023.1 | 21 | 0.013 | **X** |
| elongation factor Tu | UXO40786.1 | 43 | < 0.00010 | **X** |
| translation elongation factor Ts | UXO41421.1 | 31 | < 0.00010 | **X** |
| elongation factor G | UXO58489.1 | 78 | < 0.00010 | **X** |
| tyrosine--tRNA ligase | UXO58474.1 | 44 | 0.025 | **X** |
| ribosome-associated translation inhibitor RaiA | WP_003094359.1 | 12 | 0.024 | **X** |
| DNA gyrase subunit A | UXO36018.1 | 101 | 0.0010 | **X** |
| DNA polymerase I | UXO45927.1 | 100 | 0.0081 | **X** |
| DNA polymerase III subunit beta | UXO40102.1 | 41 | 0.00097 | **X** |
| DNA-binding protein HU-beta | UXO37563.1 | 9 | 0.016 | **X** |
| exodeoxyribonuclease III | UXO57572.1 | 30 | 0.0047 | **X** |
| single-stranded DNA-binding protein | UXO34918.1 | 19 | 0.00029 | **X** |
| polyribonucleotide nucleotidyltransferase | UXO45180.1 | 75 | 0.00014 | **X** |
| chaperone surA | EOT10411.1 | 48 | < 0.00010 | **X** |
| molecular chaperone DnaK | UXO57004.1 | 68 | < 0.00010 | **X** |
| EVE domain-containing protein | UXO39767.1 | 17 | < 0.00010 | **X** |
| trigger factor | UXO43466.1 | 49 | 0.00059 | **X** |
| anti-virulence regulator CigR family protein | WP_003139043.1 | 17 | 0.0021 | **X** |
| quorum-sensing-regulated virulence factor family protein QSregVF | UXO47364.1 | 15 | < 0.00010 | **X** |
| YajQ family cyclic di-GMP-binding protein | UXO56528.1 | 18 | < 0.00010 | **X** |
| diguanylate cyclase inhibitor YfiR | UXO38254.1 | 21 | 0.014 | **X** |
| carbon storage regulator CsrA | UXO44367.1 | 7 | 0.00021 | **X** |
| transcriptional regulator MvaU | UXO54225.1 | 13 | 0.0024 | **X** |
| BolA family transcriptional regulator | UXO56582.1 | 9 | 0.026 | **X** |
| **30S and 50S ribosomal proteins** | | | | |
| 30S ribosomal protein S11 | UXO34910.1 | 14 | 0.016 | **X** |
| 30S ribosomal protein S13 | UXO52612.1 | 13 | < 0.00010 | **X** |
| 30S ribosomal protein S14 | UXO64406.1 | 12 | 0.0011 | **X** |
| 30S ribosomal protein S15 | EOT07060.1 | 10 | <0.00010 | **X** |
| 30S ribosomal protein S16 | UXO59032.1 | 9 | < 0.00010 | **X** |
| 30S ribosomal protein S18 | UXO51271.1 | 9 | < 0.00010 | **X** |
| 30S ribosomal protein S19 | UXO46693.1 | 10 | < 0.00010 | **X** |
| 30S ribosomal protein S2 | UXO35517.1 | 27 | < 0.00010 | **X** |
| 30S ribosomal protein S3 | UXO52596.1 | 26 | < 0.00010 | **X** |
| 30S ribosomal protein S4 | UXO46713.1 | 23 | 0.00016 | **X** |
| 30S ribosomal protein S5 | WP_003093697.1 | 18 | < 0.00010 | **X** |
| 30S ribosomal protein S7 | UXO64389.1 | 18 | 0.0037 | **X** |
| 30S ribosomal protein S8 | UXO64407.1 | 14 | 0.015 | **X** |
| 50S ribosomal protein L1 | UXO46679.1 | 24 | < 0.00010 | **X** |
| 50S ribosomal protein L10 | UXO52581.1 | 18 | 0.0013 | **X** |
| 50S ribosomal protein L13 | EOT22056.1 | 16 | 0.00033 | **X** |
| 50S ribosomal protein L14 | UXO52600.1 | 13 | < 0.00010 | **X** |
| 50S ribosomal protein L15 | WP_003093695.1 | 15 | 0.0076 | **X** |
| 50S ribosomal protein L16 | UXO46696.1 | 15 | < 0.00010 | **X** |
| 50S ribosomal protein L17 | UXO34913.1 | 15 | < 0.00010 | **X** |
| 50S ribosomal protein L18 | UXO52606.1 | 13 | < 0.00010 | **X** |
| 50S ribosomal protein L19 | UXO47233.1 | 13 | < 0.00010 | **X** |
| 50S ribosomal protein L2 | WP_003103878.1 | 30 | < 0.00010 | **X** |
| 50S ribosomal protein L22 | UXO46694.1 | 12 | < 0.00010 | **X** |
| 50S ribosomal protein L23 | WP_003093736.1 | 11 | 0.0018 | **X** |
| 50S ribosomal protein L24 | UXO34898.1 | 11 | 0.00033 | **X** |
| 50S ribosomal protein L25 | UXO39209.1 | 22 | < 0.00010 | **X** |
| 50S ribosomal protein L27 | UXO62709.1 | 9 | 0.0033 | **X** |
| 50S ribosomal protein L28 | UXO51657.1 | 9 | 0.00030 | **X** |
| 50S ribosomal protein L29 | UXO34895.1 | 7 | 0.012 | **X** |
| 50S ribosomal protein L30 | UXO40806.1 | 6 | < 0.00010 | **X** |
| 50S ribosomal protein L31 | UXO63189.1 | 8 | 0.025 | **X** |
| 50S ribosomal protein L31 type B | EOT11293.1 | 10 | < 0.00010 | **X** |
| 50S ribosomal protein L32 | UXO65709.1 | 7 | 0.0033 | **X** |
| 50S ribosomal protein L4 | UXO40789.1 | 22 | 0.013 | **X** |
| 50S ribosomal protein L5 | UXO40800.1 | 20 | < 0.00010 | **X** |
| 50S ribosomal protein L6 | UXO40803.1 | 19 | < 0.00010 | **X** |
| 50S ribosomal protein L9 | UXO39467.1 | 16 | < 0.00010 | **X** |

*Quantitative profile

**X** - Downregulated in double mutant in comparison to wild type and rescued to wild type-status when AHL added.

**X** - Upregulated in double mutant in comparison to wild type and rescued to wild type-status when AHL added.

**X** - Upregulated in double mutant in comparison to wild type.

**X** - No changes in double mutant in comparison to wild type. Distinct effect of AHL in either wild type or double mutant or both.

**Table S3.** Selected groups of proteins differentially expressed in wild-type *P. aeruginosa* PA14 and its *lasI-/rhlI-* mutant exposed to AHL mixture or treated with DMSO as a diluent control; the proteins are overall allocated to the functional group **transport and secretion systems** according to NCBI GO analyses.

| **Identified proteins** | **NCBI**  **accession**  **number** | **MW**  **kDa** | ***p*-value** | **Quantitative profile *** |
| --- | --- | --- | --- | --- |
| **Transport and secretion systems** | | | | |
| **ABC and TRAP transporters** | | | | |
| ABC amino acid transporter | EOT07981.1 | 29 | 0.00061 | **X** |
| ABC transporter substrate-binding protein | UXO64804.1 | 34 | < 0.00010 | **X** |
|  | EOT17153.1 | 29 | < 0.00010 | **X** |
|  | EOT21988.1 | 60 | < 0.00010 | **X** |
|  | UXO42491.1 | 34 | < 0.00010 | **X** |
|  | UXO63278.1 | 28 | < 0.00010 | **X** |
|  | UXO39639.1 | 38 | < 0.00010 | **X** |
|  | UXO57560.1 | 58 | < 0.00010 | **X** |
|  | UXO51493.1 | 28 | < 0.00010 | **X** |
|  | EOT21992.1 | 60 | 0.00013 | **X** |
|  | UXO42829.1 | 37 | 0.00018 | **X** |
|  | UXO58415.1 | 38 | 0.00034 | **X** |
|  | UXO32584.1 | 28 | 0.0013 | **X** |
|  | EOT06718.1 | 30 | 0.0018 | **X** |
|  | UXO59865.1 | 35 | 0.0020 | **X** |
|  | UXO45576.1 | 28 | 0.0097 | **X** |
|  | UXO50725.1 | 59 | 0.018 | **X** |
|  | UXO52971.1 | 28 | < 0.00010 | **X** |
|  | UXO62534.1 | 59 | < 0.00010 | **X** |
| aliphatic sulfonate ABC transporter substrate-binding protein | UXO59312.1 | 36 | 0.0043 | **X** |
|  | UXO66098.1 | 35 | < 0.00010 | **X** |
| branched-chain amino acid ABC transporter substrate-binding protein | UXO57153.1 | 40 | 0.0035 | **X** |
|  | UXO44202.1 | 40 | < 0.00010 | **X** |
| choline ABC transporter substrate-binding protein | UXO51714.1 | 34 | < 0.00010 | **X** |
| cystine ABC transporter substrate-binding protein | UXO58126.1 | 28 | < 0.00010 | **X** |
| glutamate/aspartate ABC transporter substrate-binding protein | UXO55721.1 | 33 | < 0.00010 | **X** |
| glycine betaine ABC transporter substrate-binding protein | UXO58850.1 | 35 | 0.00031 | **X** |
| glycine betaine ABC transporter substrate-binding protein | UXO65452.1 | 31 | 0.0056 | **X** |
| Fe2+-enterobactin ABC transporter substrate-binding protein | UXO52692.1 | 32 | < 0.00010 | **X** |
| hemin ABC transporter substrate-binding protein | UXO45148.1 | 31 | < 0.00010 | **X** |
| iron ABC transporter substrate-binding protein | UXO51027.1 | 36 | < 0.00010 | **X** |
| MetQ/NlpA family ABC transporter substrate-binding protein | UXO63642.1 | 28 | 0.00021 | **X** |
| MetQ/NlpA family ABC transporter substrate-binding protein | UXO58807.1 | 28 | < 0.00010 | **X** |
| molybdate ABC transporter, periplasmic molybdate-binding protein | EOT186 | 26 | < 0.00010 | **X** |
| phosphate ABC transporter substrate-binding protein PstS | UXO34002.1 | 34 | < 0.00010 | **X** |
| putative selenate ABC transporter substrate-binding protein | UXO59449.1 | 31 | < 0.00010 | **X** |
| sugar ABC transporter substrate-binding protein | UXO37422.1 | 34 | < 0.00010 | **X** |
| sulfate ABC transporter substrate-binding protein | WP_003084243.1 | 36 | 0.0053 | **X** |
|  | UXO49676.1 | 37 | < 0.00010 | **X** |
| sulfonate ABC transporter substrate-binding protein | UXO47514.1 | 35 | < 0.00010 | **X** |
| taurine ABC transporter substrate-binding protein | UXO52899.1 | 36 | < 0.00010 | **X** |
| zinc ABC transporter substrate-binding protein | UXO63636.1 | 34 | 0.00069 | **X** |
| C4-dicarboxylate TRAP substrate-binding protein DctP | UXO33804.1 | 37 | < 0.00010 | **X** |
| c4-dicarboxylate-binding protein | EOT21765.1 | 37 | < 0.00010 | **X** |
| TAXI family TRAP transporter solute-binding subunit | UXO34174.1 | 34 | 0.00051 | **X** |
| TRAP transporter substrate-binding protein DctP | UXO35359.1 | 38 | 0.0025 | **X** |
| transporter substrate-binding domain-containing protein | UXO35114.1 | 33 | < 0.00010 | **X** |
|  | UXO51419.1 | 33 | < 0.00010 | **X** |
|  | UXO43662.1 | 35 | < 0.00010 | **X** |
|  | UXO46895.1 | 28 | 0.012 | **X** |
| extracellular solute-binding protein | UXO46332.1 | 39 | 0.0091 | **X** |
|  | UXO45656.1 | 36 | < 0.00010 | **X** |
|  | UXO49756.1 | 40 | 0.014 | **X** |
|  | UXO35914.1 | 38 | 0.00046 | **X** |
| tripartite tricarboxylate transporter substrate binding protein | UXO62229.1 | 35 | 0.0016 | **X** |
| putrescine-binding protein SpuD | UXO52210.1 | 41 | < 0.00010 | **X** |
| spermidine-binding protein SpuE | WP_003084301.1 | 40 | < 0.00010 | **X** |
| **Porins, efflux and outer membrane proteins** | | | | |
| membrane integrity-associated transporter subunit PqiC | UXO51299.1 | 26 | 0.016 | **X** |
| efflux RND transporter periplasmic adaptor subunit | UXO51499.1 | 43 | 0.0076 | **X** |
| efflux transporter outer membrane subunit | UXO51498.1 | 53 | 0.014 | **X** |
| outer membrane ferric siderophore receptor | EOT21974.1 | 82 | 0.0055 | **X** |
| outer membrane protein assembly factor BamE | WP_003109323.1 | 19 | 0.012 | **X** |
| outer membrane protein OprG | UXO64582.1 | 25 | 0.0018 | **X** |
| outer membrane protein transport protein | UXO32170.1 | 46 | 0.011 | **X** |
| outer membrane protein, OmpA | EOT11384.1 | 29 | 0.00016 | **X** |
| Co2+/Mg2+ efflux protein ApaG | UXO34798.1 | 14 | 0.0012 | **X** |
| lipopolysaccharide transport periplasmic protein LptA | UXO62493.1 | 19 | 0.00023 | **X** |
| OmpA family protein | UXO56241.1 | 25 | < 0.00010 | **X** |
| OmpA family protein | UXO44229.1 | 32 | 0.00012 | **X** |
| OprD family porin | UXO58103.1 | 50 | < 0.00010 | **X** |
| phospholipid-binding protein MlaC | UXO32979.1 | 24 | 0.00018 | **X** |
| **Secretion system proteins** | | | | |
| alkaline protease secretion protein AprF | UXO44011.1 | 54 | 0.0052 | **X** |
| general secretion pathway protein G | EOT14837.1 | 15 | 0.0020 | **X** |
| secretin | UXO42458.1 | 29 | < 0.00010 | **X** |
| type 4a pilus secretin PilQ | UXO33674.1 | 77 | < 0.00010 | **X** |
| type VI secretion system contractile sheath large subunit | UXO57888.1 | 56 | < 0.00010 | **X** |
| type VI secretion system receptor/chaperone Hcp | UXO63790.1 | 17 | 0.00033 | **X** |
| type VI secretion system-associated lipoprotein TagQ | UXO57874.1 | 32 | < 0.00010 | **X** |
| **TonB transporters** | | | | |
| TonB-dependent copper receptor | UXO58952.1 | 79 | 0.0014 | **X** |
| TonB-dependent receptor | UXO38597.1 | 74 | 0.014 | **X** |
|  | UXO49242.1 | 72 | 0.0041 | **X** |
|  | UXO51016.1 | 81 | 0.00099 | **X** |
|  | UXO34991.1 | 77 | 0.014 | **X** |
| protein tonB | EOT09914.1 | 37 | < 0.00010 | **X** |
| **Iron uptake and metal ion binding** | | | | |
| ferrioxamine receptor FoxA | UXO60369.1 | 90 | 0.00011 | **X** |
| ferripyoverdine/pyocin S3 receptor FpvA | UXO66346.1 | 91 | < 0.00010 | **X** |
| heme/hemoglobin uptake receptor PhuR | UXO56953.1 | 85 | 0.0036 | **X** |
| type I ferripyoverdine receptor, FpvB | EOT11907.1 | 87 | 0.0018 | **X** |
| FecR family protein | UXO52380.1 | 36 | 0.0014 | **X** |
| copper chaperone PCu(A)C | UXO64858.1 | 17 | 0.00051 | **X** |
| mercury resistance system periplasmic binding protein MerP | UXO53084.1 | 9 | 0.0031 | **X** |

*Quantitative profile

**X** - Downregulated in double mutant in comparison to wild type and rescued to wild type-status when AHL added.

**X** - Upregulated in double mutant in comparison to wild type and rescued to wild type-status when AHL added.

**X** - Upregulated in double mutant in comparison to wild type.

**X** - No changes in double mutant in comparison to wild type. Distinct effect of AHL in either wild type or double mutant or both.

**Table S4.** Selected groups of proteins differentially expressed in wild-type *P. aeruginosa* PA14 and its *lasI-/rhlI-* mutant exposed to AHL mixture or treated with DMSO as a diluent control; the proteins are overall allocated to the functional group **cell envelope integrity** according to NCBI GO analyses.

| **Identified proteins** | **NCBI accession number** | **MW kDa** | ***p*-value** | **Quantitative profile *** |
| --- | --- | --- | --- | --- |
| **Cell envelope integrity** | | | | |
| **Tol-Pal system for envelope maintenance (outer membrane – peptidoglycan tethering)** | | | | |
| Tol-Pal system beta propeller repeat protein TolB | UXO32500.1 | 48 | < 0.00010 | **X** |
| tol-pal system protein YbgF | UXO50200.1 | 29 | < 0.00010 | **X** |
| **Envelope regulation and adaptation** | | | | |
| YgdI/YgdR family lipoprotein | UXO42685.1 | 8 | 0.0096 | **X** |
| carbon storage regulator CsrA | UXO44367.1 | 7 | 0.00021 | **X** |
| BolA family transcriptional regulator | UXO56582.1 | 9 | 0.026 | **X** |
| **Outer membrane structure and assembly** | | | | |
| outer membrane lipoprotein chaperone LolA | WP_003090414.1 | 23 | < 0.00010 | **X** |
| outer membrane protein assembly factor BamE | WP_003109323.1 | 19 | 0.012 | **X** |
| skp-like protein | EOT11339.1 | 19 | < 0.00010 | **X** |
| chaperone surA | EOT10411.1 | 48 | < 0.00010 | **X** |
| outer membrane protein OprG | UXO64582.1 | 25 | 0.0018 | **X** |
| outer membrane protein transport protein | UXO32170.1 | 46 | 0.011 | **X** |
| outer membrane protein, OmpA | EOT11384.1 | 29 | 0.00016 | **X** |
| OmpA family protein | UXO56241.1 | 25 | < 0.00010 | **X** |
|  | UXO44229.1 | 32 | 0.00012 | **X** |
| OprD family porin | UXO58103.1 | 50 | < 0.00010 | **X** |
| lysin domain-containing protein | EOT09855.1 | 38 | < 0.00010 | **X** |
| LPS assembly lipoprotein LptE | UXO64657.1 | 23 | 0.00079 | **X** |
| lipopolysaccharide transport periplasmic protein LptA | UXO62493.1 | 19 | 0.00023 | **X** |
| peptidoglycan-associated lipoprotein | EOT20697.1 | 18 | 0.0029 | **X** |
| **Envelope synthesis, support and remodeling** | | | | |
| UDP-N-acetyl-D-mannosamine dehydrogenase | UXO41932.1 | 46 | 0.00067 | **X** |
| UDP-N-acetylglucosamine 2-epimerase (non-hydrolyzing) | UXO65534.1 | 42 | 0.00025 | **X** |
| UDP-N-acetylmuramoyl-L-alanyl-D-glutamate--2,6-diaminopimelate ligase | UXO50648.1 | 51 | 0.00061 | **X** |
| UDP-N-acetylmuramoyl-tripeptide--D-alanyl-D-alanine ligase | UXO38845.1 | 47 | 0.024 | **X** |
| N-acetylmuramoyl-L-alanine amidase AmpDh3 | UXO32669.1 | 29 | < 0.00010 | **X** |
| N-acetylmuramoyl-L-alanine amidase AmiB | UXO51283.1 | 51 | < 0.00010 | **X** |
| L,D-transpeptidase family protein | UXO65822.1 | 35 | < 0.00010 | **X** |
| penicillin-binding protein 2 | WP_003110751.1 | 61 | 0.0012 | **X** |
| peptidoglycan binding protein CsiV | UXO65676.1 | 22 | < 0.00010 | **X** |
| peptidoglycan DD-metalloendopeptidase family protein | UXO47352.1 | 31 | 0.013 | **X** |
|  | UXO41253.1 | 31 | < 0.00010 | **X** |
| peptidoglycan-binding protein LysM | UXO33815.1 | 15 | 0.00015 | **X** |
| lipoprotein | EOT10980.1 | 23 | 0.026 | **X** |
|  | UXO39817.1 | 5 | 0.00051 | **X** |
|  | EOT22247.1 | 29 | < 0.00010 | **X** |
| SPOR domain-containing protein | WP_003112013.1 | 26 | < 0.00010 | **X** |
| lytic murein transglycosylase | WP_003140529.1 | 43 | < 0.00010 | **X** |
| lytic murein transglycosylase | WP_003118255.1 | 48 | 0.00019 | **X** |
| lytic murein transglycosylase B | EOT11736.1 | 37 | 0.0052 | **X** |
| membrane-bound lytic murein transglycosylase A | EOT20962.1 | 42 | 0.014 | **X** |
| acetyl-CoA C-acyltransferase | UXO64713.1 | 41 | 0.0026 | **X** |
| acetyl-CoA carboxylase biotin carboxylase subunit | UXO62990.1 | 49 | 0.018 | **X** |
| enoyl-ACP reductase FabV | UXO59828.1 | 44 | 0.00012 | **X** |
| 3-oxoacyl-ACP reductase FabG | UXO59811.1 | 26 | 0.018 | **X** |
| beta-ketoacyl-ACP synthase FabY | UXO51514.1 | 69 | < 0.00010 | **X** |
| bifunctional UDP-N-acetylglucosamine diphosphorylase/  glucosamine-1-phosphate N-acetyltransferase GlmU | UXO45983.1 | 49 | 0.013 | **X** |
| phosphoheptose isomerase | UXO38854.1 | 21 | 0.0011 | **X** |
| phosphomannomutase/phosphoglucomutase | UXO51662.1 | 92 | < 0.00010 | **X** |
| acyl carrier protein | UXO42110.1 | 9 | 0.024 | **X** |
| **Defense and host interaction** | | | | |
| lysozyme inhibitor Ivy | UXO41136.1 | 17 | < 0.00010 | **X** |
| inhibitor of vertebrate lysozyme family protein | UXO51816.1 | 17 | < 0.00010 | **X** |
| lysozyme inhibitor LprI family protein | UXO47956.1 | 14 | 0.0053 | **X** |
| **Other** | | | | |
| MaoC family dehydratase | UXO46929.1 | 17 | 0.0018 | **X** |
| ATP-dependent zinc protease | UXO58272.1 | 19 | 0.00029 | **X** |
| ATP-dependent zinc protease | WP_003087811.1 | 20 | < 0.00010 | **X** |
| gamma-glutamyltransferase | WP_003086881.1 | 60 | < 0.00010 | **X** |
| OXA-50 family oxacillin-hydrolyzing class D beta-lactamase OXA-488 | UXO63650.1 | 29 | 0.00027 | **X** |
| oxidoreductase | UXO58729.1 | 23 | 0.00043 | **X** |
| bifunctional protein-disulfide isomerase/oxidoreductase DsbC | UXO35435.1 | 26 | 0.026 | **X** |
| protein-methionine-sulfoxide reductase catalytic subunit MsrP | UXO45131.1 | 38 | 0.0096 | **X** |
| thiol peroxidase | UXO54402.1 | 17 | < 0.00010 | **X** |
| transglutaminase family protein | UXO31422.1 | 70 | 0.00017 | **X** |
| phosphoglycerate dehydrogenase | UXO40424.1 | 44 | 0.0029 | **X** |
| 3-deoxy-8-phosphooctulonate synthase | UXO35537.1 | 31 | < 0.00010 | **X** |
| esterase EstA | UXO57352.1 | 70 | < 0.00010 | **X** |
| esterase family protein | UXO54487.1 | 58 | 0.0064 | **X** |
| esterase TesA | UXO42218.1 | 21 | < 0.00010 | **X** |
| gamma-glutamyltransferase | UXO34569.1 | 62 | 0.0011 | **X** |
| glucan biosynthesis protein G | UXO33712.1 | 59 | 0.0047 | **X** |
| glycerophosphodiester phosphodiesterase | UXO48691.1 | 41 | 0.011 | **X** |

*Quantitative profile

**X** - Downregulated in double mutant in comparison to wild type and rescued to wild type-status when AHL added.

**X** - Upregulated in double mutant in comparison to wild type and rescued to wild type-status when AHL added.

**X** - Upregulated in double mutant in comparison to wild type.

**X** - No changes in double mutant in comparison to wild type. Distinct effect of AHL in either wild type or double mutant or both.

**Table S5.** Selected groups of proteins differentially expressed in wild-type *P. aeruginosa* PA14 and its *lasI-/rhlI-* mutant exposed to AHL mixture or treated with DMSO as a diluent control; the proteins are overall allocated to the functional group **redox processes** according to NCBI GO analyses.

| **Identified proteins** | **NCBI accession number** | **MW kDa** | ***p*-value** | **Quantitative profile*** |
| --- | --- | --- | --- | --- |
| **Redox processes** | | | | |
| **Electron and ion transfer, redox processes and stress response** | | | | |
| azurin | UXO45359.1 | 16 | < 0.00010 | **X** |
| cytochrome c oxidase, cbb3-type, subunit II | EOT21296.1 | 23 | < 0.00010 | **X** |
| cytochrome c5 | EOT07951.1 | 14 | 0.0031 | **X** |
| cytochrome c5 family protein | UXO51826.1 | 10 | 0.00072 | **X** |
| cytochrome-c oxidase, cbb3-type subunit III | WP_003087308.1 | 34 | < 0.00010 | **X** |
| ferredoxin-NADP reductase | WP_003091832.1 | 30 | < 0.00010 | **X** |
| flavodoxin | UXO35722.1 | 16 | 0.00042 | **X** |
| ISC system 2Fe-2S type ferredoxin | UXO35328.1 | 12 | 0.0016 | **X** |
| electron transfer flavoprotein subunit beta/FixA family protein | UXO53924.1 | 26 | 0.00080 | **X** |
| Grx4 family monothiol glutaredoxin | UXO47441.1 | 12 | < 0.00010 | **X** |
| thioredoxin TrxA | UXO39778.1 | 12 | 0.0088 | **X** |
| thioredoxin reductase | EOT14322.1 | 34 | 0.00016 | **X** |
| YceI family protein | UXO38773.1 | 22 | 0.0037 | **X** |
|  | UXO58236.1 | 21 | 0.014 | **X** |
| F0F1 ATP synthase subunit alpha | UXO40086.1 | 55 | 0.0039 | **X** |
| F0F1 ATP synthase subunit B | UXO63693.1 | 17 | 0.0015 | **X** |
| F0F1 ATP synthase subunit beta | UXO63689.1 | 50 | < 0.00010 | **X** |
| F0F1 ATP synthase subunit delta | UXO63692.1 | 19 | 0.014 | **X** |
| F0F1 ATP synthase subunit epsilon | UXO40083.1 | 15 | 0.024 | **X** |
| Fe2+-dependent dioxygenase | UXO37180.1 | 26 | < 0.00010 | **X** |
| ferrioxamine receptor FoxA | UXO60369.1 | 90 | 0.00011 | **X** |
| Fe-S cluster assembly protein IscX | UXO47132.1 | 8 | 0.0014 | **X** |
| Fe-S cluster assembly scaffold IscU | UXO58929.1 | 14 | < 0.00010 | **X** |
| succinate dehydrogenase flavoprotein subunit | EOT21323.1 | 64 | < 0.00010 | **X** |
| SDR family oxidoreductase | UXO44719.1 | 27 | < 0.00010 | **X** |
| superoxide dismutase | EOT22124.1 | 21 | 0.018 | **X** |
| thiol peroxidase | UXO54402.1 | 17 | < 0.00010 | **X** |
| alkyl hydroperoxide reductase subunit C | UXO40239.1 | 21 | 0.0014 | **X** |
| ArsC family reductase | UXO41412.1 | 13 | < 0.00010 | **X** |
| arsenate reductase (glutaredoxin) | UXO62028.1 | 13 | 0.00065 | **X** |
| glutathione peroxidase | UXO61678.1 | 20 | < 0.00010 | **X** |
| glutathione-disulfide reductase | WP_003139698.1 | 49 | 0.0014 | **X** |
| NAD(P)/FAD-dependent oxidoreductase | UXO40873.1 | 33 | 0.018 | **X** |
| NADP(H)-dependent aldo-keto reductase | UXO50665.1 | 39 | 0.00088 | **X** |
| NADP-specific glutamate dehydrogenase | UXO33224.1 | 49 | 0.014 | **X** |
| Si-specific NAD(P)(+) transhydrogenase | UXO65687.1 | 51 | 0.00066 | **X** |
| nitroreductase family protein | UXO54317.1 | 22 | 0.0068 | **X** |
| nitrous oxide reductase accessory protein NosL | UXO35761.1 | 19 | 0.0081 | **X** |
| oxidoreductase | UXO58729.1 | 23 | 0.00043 | **X** |
| protein-methionine-sulfoxide reductase catalytic subunit MsrP | UXO45131.1 | 38 | 0.0096 | **X** |
| ribonucleoside-diphosphate reductase subunit alpha | UXO32319.1 | 107 | 0.0040 | **X** |
| oxidative damage protection protein | UXO57390.1 | 11 | 0.012 | **X** |
| peroxiredoxin C | UXO47445.1 | 22 | < 0.00010 | **X** |

*Quantitative profile

**X** - Downregulated in double mutant in comparison to wild type and rescued to wild type-status when AHL added.

**X** - Upregulated in double mutant in comparison to wild type and rescued to wild type-status when AHL added.

**X** - Upregulated in double mutant in comparison to wild type.

**X** - No changes in double mutant in comparison to wild type. Distinct effect of AHL in either wild type or double mutant or both.

**Table S6.** Selected groups of proteins differentially expressed in wild-type *P. aeruginosa* PA14 and its *lasI-/rhlI-* mutant exposed to AHL mixture or treated with DMSO as a diluent control; the proteins are overall allocated to the following functional groups: **motility**; **invasiveness and toxicity**; and **enzyme dependent antibiotic resistance** according to NCBI GO analyses.

| **Identified proteins** | **NCBI accession number** | **MW kDa** | ***p*-value** | **Quantitative profile *** |
| --- | --- | --- | --- | --- |
| **Motility** | | | | |
| flagellar basal-body rod protein FlgF | UXO61902.1 | 27 | 0.026 | **X** |
| flagella basal body P-ring formation protein FlgA | EOT15105.1 | 27 | 0.0039 | **X** |
| flagellar assembly peptidoglycan hydrolase FlgJ | UXO32391.1 | 43 | 0.0090 | **X** |
| flagellar basal body P-ring protein FlgI | UXO61899.1 | 38 | 0.0050 | **X** |
| flagellar biosynthesis anti-sigma factor FlgM | UXO65312.1 | 11 | < 0.00010 | **X** |
| flagellar basal body-associated protein FliL | WP_003096328.1 | 15 | 0.00053 | **X** |
| flagellar filament capping protein FliD | WP_003140595.1 | 49 | 0.00017 | **X** |
| flagellar hook assembly protein FlgD | UXO38298.1 | 25 | 0.0024 | **X** |
| flagellar hook protein FlgE | UXO32396.1 | 48 | 0.016 | **X** |
| flagellar hook-basal body complex protein FliE | UXO55979.1 | 12 | < 0.00010 | **X** |
| flagellar protein FlaG | UXO44183.1 | 13 | < 0.00010 | **X** |
| chemotaxis protein CheW | UXO61510.1 | 18 | 0.0031 | **X** |
| chemotaxis transducer | EOT21968.1 | 72 | 0.0052 | **X** |
| chemotaxis response regulator protein-glutamate methylesterase | WP_003087115.1 | 39 | 0.0015 | **X** |
| type 1 fimbrial protein | UXO33829.1 | 13 | 0.0041 | **X** |
| type 4a pilus biogenesis protein PilF | UXO58937.1 | 29 | < 0.00010 | **X** |
| type 4a pilus secretin PilQ | UXO33674.1 | 77 | < 0.00010 | **X** |
| PilY1 | AAP81276.1 | 128 | 0.0022 | **X** |
| **Invasiveness and toxicity** | | | | |
| serralysin family metalloprotease AprA | WP_003086731.1 | 50 | < 0.00010 | **X** |
| ImpA family metalloprotease | WP_003137348.1 | 100 | 0.0014 | **X** |
| elastinolytic metalloprotease LasA | UXO55196.1 | 46 | 0.0064 | **X** |
| alkaline protease secretion protein AprF | UXO44011.1 | 54 | 0.0052 | **X** |
| M4 family elastase LasB | UXO47252.1 | 54 | 0.0036 | **X** |
| chitin-binding protein CbpD | UXO62126.1 | 42 | 0.017 | **X** |
| T3SS effector bifunctional cytotoxin exoenzyme T | UXO51947.1 | 47 | 0.0057 | **X** |
| alkaline proteinase inhibitor AprI | UXO49910.1 | 14 | 0.00098 | **X** |
| aminopeptidase PaaP | UXO65740.1 | 58 | 0.0051 | **X** |
| esterase EstA | UXO57352.1 | 70 | < 0.00010 | **X** |
| esterase family protein | UXO54487.1 | 58 | 0.0064 | **X** |
| hemagglutinin repeat-containing protein | WP_016254199.1 | 532 | < 0.00010 | **X** |
| methionine aminopeptidase, type I | EOT11349.1 | 29 | 0.0021 | **X** |
| periplasmic tail-specific protease | EOT15007.1 | 79 | 0.0031 | **X** |
| M14 family metallopeptidase | UXO42023.1 | 71 | 0.0012 | **X** |
| neutral zinc metallopeptidase | UXO51058.1 | 32 | 0.018 | **X** |
| YicC family protein | UXO57575.1 | 32 | < 0.00010 | **X** |
| protease IV | UXO58572.1 | 48 | < 0.00010 | **X** |
| **Enzyme dependent antibiotic resistance** | | | | |
| protease PfpI | UXO34563.1 | 19 | 0.013 | **X** |
| OXA-50 family oxacillin-hydrolyzing class D beta-lactamase OXA-488 | UXO63650.1 | 29 | 0.00027 | **X** |
| DNA gyrase subunit A | UXO36018.1 | 101 | 0.0010 | **X** |

*Quantitative profile

**X** - Downregulated in double mutant in comparison to wild type and rescued to wild type-status when AHL added.

**X** - Upregulated in double mutant in comparison to wild type and rescued to wild type-status when AHL added.

**X** - Upregulated in double mutant in comparison to wild type.

**X** - No changes in double mutant in comparison to wild type. Distinct effect of AHL in either wild type or double mutant or both.
